# Selection for Protein Kinetic Stability Connects Denaturation Temperatures to Organismal Temperatures and Provides Clues to Archaean Life

**DOI:** 10.1371/journal.pone.0156657

**Published:** 2016-06-02

**Authors:** M. Luisa Romero-Romero, Valeria A. Risso, Sergio Martinez-Rodriguez, Eric A. Gaucher, Beatriz Ibarra-Molero, Jose M. Sanchez-Ruiz

**Affiliations:** 1 Departamento de Quimica Fisica, Facultad de Ciencias, Universidad de Granada, 18071, Granada, Spain; 2 Georgia Institute of Technology, School of Biology, School of Chemistry and Biochemistry, and Parker H. Petit Institute for Bioengineering and Biosciences, Atlanta, Georgia, 30332, United States of America; University of Colorado Anschutz Medical Campus, UNITED STATES

## Abstract

The relationship between the denaturation temperatures of proteins (T_m_ values) and the living temperatures of their host organisms (environmental temperatures: T_ENV_ values) is poorly understood. Since different proteins in the same organism may show widely different T_m_’s, no simple universal relationship between T_m_ and T_ENV_ should hold, other than T_m_≥T_ENV_. Yet, when analyzing a set of homologous proteins from different hosts, T_m_’s are oftentimes found to correlate with T_ENV_’s but this correlation is shifted upward on the T_m_ axis. Supporting this trend, we recently reported T_m_’s for resurrected Precambrian thioredoxins that mirror a proposed environmental cooling over long geological time, while remaining a shocking ~50°C above the proposed ancestral ocean temperatures. Here, we show that natural selection for protein kinetic stability (denaturation rate) can produce a T_m_↔T_ENV_ correlation with a large upward shift in T_m_. A model for protein stability evolution suggests a link between the T_m_ shift and the *in vivo* lifetime of a protein and, more specifically, allows us to estimate ancestral environmental temperatures from experimental denaturation rates for resurrected Precambrian thioredoxins. The T_ENV_ values thus obtained match the proposed ancestral ocean cooling, support comparatively high Archaean temperatures, and are consistent with a recent proposal for the environmental temperature (above 75°C) that hosted the last universal common ancestor. More generally, this work provides a framework for understanding how features of protein stability reflect the environmental temperatures of the host organisms.

## Introduction

### The temperature of ancient life

There has been considerable debate whether early life was (hyper)thermophilic or mesophilic [1]. The controversy is to a large extent linked to conjectures about the conditions under which life developed in the primitive Earth. Several scenarios [2] are consistent with the (hyper)thermophilic hypothesis, including that primordial life thrived in hydrothermal vent systems on the ocean floor, or that only resilient thermophilic organisms survived extraterrestrial catastrophic impact events (‘impact bottleneck’ scenarios) or, simply, that the late-Hadean/Archaean oceans that hosted life were hot. Indeed, oxygen and silicon isotopic composition in cherts has been taken as evidence of long-term cooling of the oceans and of comparatively high (55–85°C) Archaean temperatures [3,4]. However, these interpretations of isotopic compositions have been challenged [5,6] and analyses of fossil raindrop imprints have been recently suggested [7] to disfavor the high levels of traditional greenhouse gasses required to explain high Archaean temperatures under a Sun that was substantially dimmer than today’s. And yet, recent work has demonstrated that N_2_ and H_2_ can in fact serve as greenhouse gases under particular conditions, and that these would have helped the early Earth retain heat from the dimmer Sun at the time [8,9].

**Fig 1:**
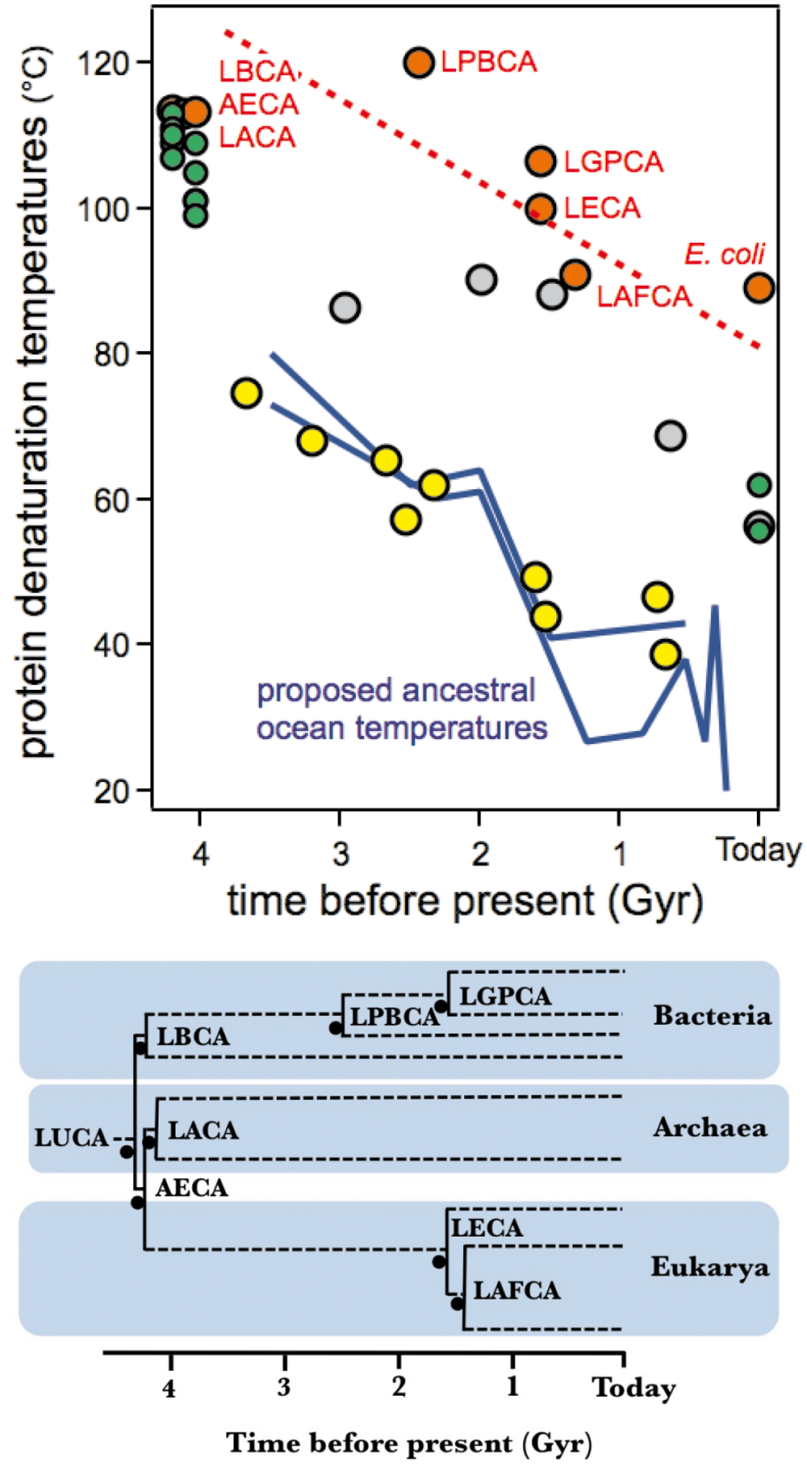
Experimental denaturation temperature values for several laboratory resurrections of ancestral proteins vs the estimated ages of the targeted Precambrian nodes. Yellow: elongation factors [10], red: thioredoxins [11], grey: ß-lactamases [12], green: nucleoside diphosphate kinases [1]. The dashed red line is meant to represent the general trend of the ancestral thioredoxins data. Estimates of the temperatures of the ancient oceans derived from the oxygen isotopic composition of cherts are also included [3, 4] as blue lines. Similar values have been calculated from silicon isotopic composition of cherts [4] but are not included for the sake of simplicity. A schematic phylogenetic tree showing the thioredoxin nodes relevant to the present work is shown.

Interestingly, recent evidence supporting the thermophilic character of early life has come from the field of experimental molecular evolution. In particular, laboratory resurrections of ancient proteins targeting Precambrian phylogenetic nodes have demonstrated substantial increases in denaturation temperature as one traverses back in time for four different gene families: elongation factors [10], thioredoxins [11], class-A β-lactamases [12] and nucleoside diphosphate kinases [1]. Each of these gene families encodes ancient proteins that have denaturation temperatures nearly 30–35 degrees higher than their modern homologs. It has been suggested that biases in the process of inferring the ancestral sequences themselves may lead to spurious increases in denaturation temperatures of the encoded proteins due to a preponderance of hydrophobic residues [13]. However, the increases in denaturation temperature obtained by ancestral protein resurrection, in particular when targeting Precambrian phylogenetic nodes are much larger than those typically obtained in protein-engineering studies aimed at enhancing protein stabilization [14] and, in fact, much larger than the estimated effects of reconstruction biases [13]. They are, therefore, hardly attributable to biases of the sequence reconstruction process, in particular since most mutations in a protein are destabilizing [15–25]. Also, we and others have demonstrated that the denaturation temperature trends are robust to uncertainties associated with inferring ancient sequences by showing that proteins encoded by several alternative reconstructed sequences for a given ancestral node display similar properties [1, 10,12]. Furthermore, plots of denaturation temperature versus geologic time (Fig 1) reveal roughly the same slope of about ten degrees per billion years for the four gene families (despite the fact that they differ in size, structure and function). Interestingly, this slope mirrors the cooling trend of the oceans proposed from the isotopic compositions of various cherts [3].

It must be noted at this point that recent ancestral resurrection work on enzymes involved in leucine biosynthesis [26] and bacterial ribonuclease H1 [27] provide evidence that local environments do not always reflect global environmental trends. Such local fluctuations may be responsible for some of the scatter observed in Fig 1. In general, however, ancient oceans were well-mixed and more homogeneous solutions because ancient circulation patterns were less disrupted by landmasses and the depth of ancient seafloors was less variable than on today’s Earth. Furthermore, the large amount of congruence observed in Fig 1 supports the idea that local environmental fluctuations do not substantially obscure the general trend that defines the change in stability between the oldest (~4 billion years) ancestors and their modern counterparts for these gene families.

### The relationship between temperatures of host organisms versus protein denaturation

**Fig 2:**
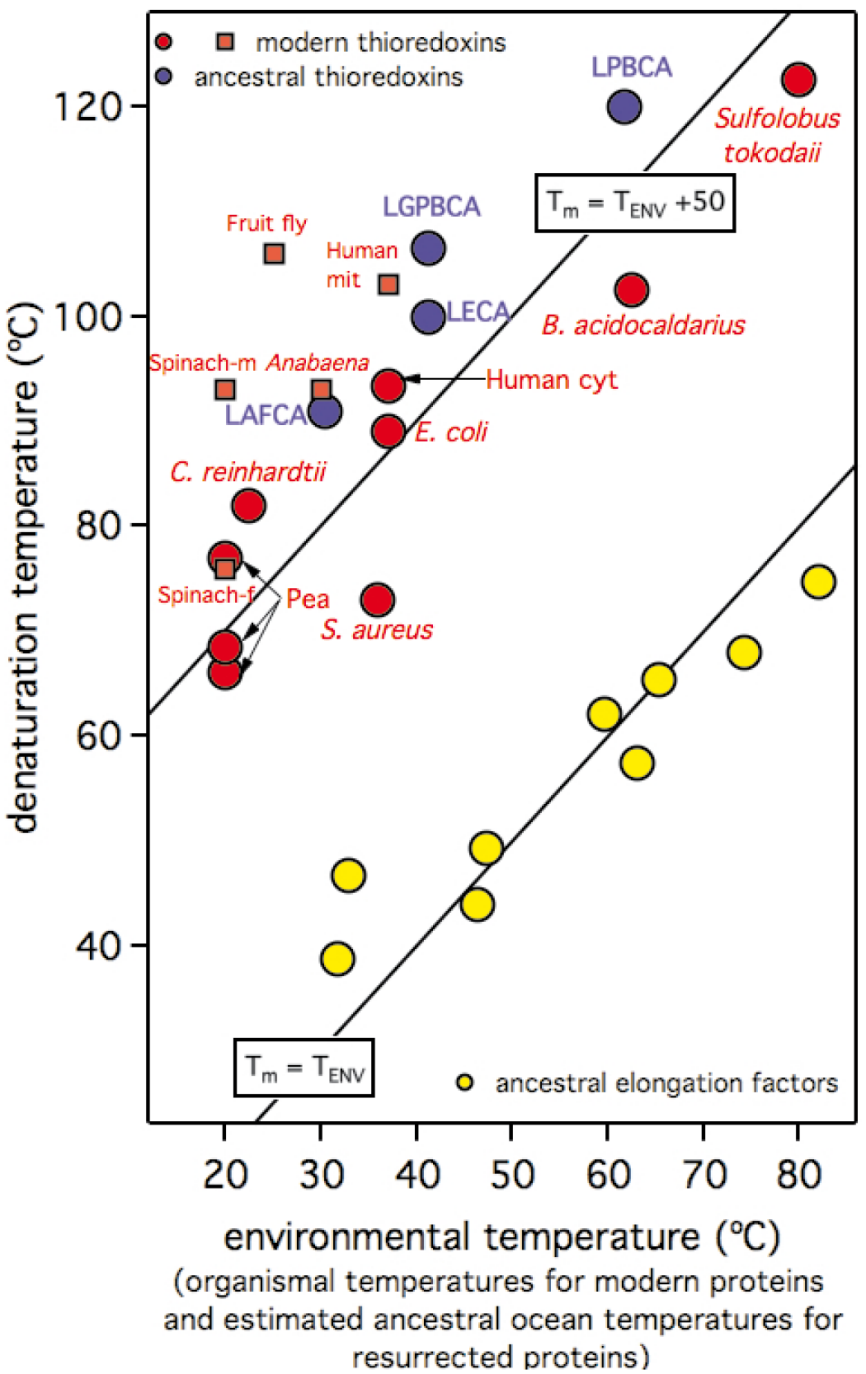
Plot of denaturation temperature *versus* environmental temperature including modern thioredoxins (red), resurrected ancestral thioredoxins (blue) and resurrected ancestral elongation factors (yellow). Elongation factor data conform approximately to the continuous line representing T_m_ = T_ENV_. Modern and ancestral thioredoxins describe a general trend that conforms a common linear dependence at about 50 degrees above the T_m_ = T_ENV_ line (the continuous line described by the equation T_m_ = T_ENV_+50 is meant to guide the eye to this common dependence). Environmental temperatures for resurrected proteins are calculated as the ancestral ocean temperatures derived from the isotopic composition of cherts (see Fig 1). Only resurrected proteins for which this calculation is possible are shown. Environmental temperatures for modern thioredoxins are the published values of the corresponding organismal growth temperatures (or the midpoints of an optimal growth range when this is provided). See "Materials and Methods" for further details on the source of the thioredoxin data in this figure. Elongation factor data are taken from [10].

The data in Fig 1 are consistent with a proposal that Archaean temperatures were higher than the average temperature today, that the Precambrian ocean cooled over a planetary time scale and that protein stability “followed” the cooling trend as host organisms adapted to changes in environmental temperatures. However, before such evidence can be accepted, it is essential to clarify a puzzling aspect of the data shown in Fig 1. Although the denaturation-temperature/geologic-time slopes are roughly the same for the four protein systems, the observed denaturation temperature values themselves are not. Elongation Factors, for instance, display denaturation temperature values that are close to the ocean temperatures proposed on the basis of isotopic composition of cherts, conversely, thioredoxins show denaturation temperatures that track ~50 degrees higher (Fig 1). Our reasons for deeming this result puzzling are expounded in detail below:

Elongation Factor Tu (EF-Tu) binds aminoacyl tRNAs and shuttles these to the ribosome. It is reasonable that EF-Tu displays a denaturation temperature value close to operating temperature since proteins that interact with nucleic acids are known to be often partially unfolded in the unbound state and to undergo a folding conformational change upon substrate binding [28–30]. Furthermore, EF-Tu undergoes additional conformational changes upon hydrolysis of GTP once the correct codon/anticodon base-pairing takes place between the mRNA and tRNA, thereby releasing the GDP-bound form from the ribosome and subsequent regeneration of the GTP-bound form through interactions with the nucleotide exchange factor [31]. We propose that the comparatively low stability of EF-Tu, as reflected in a denaturation temperature close to the environmental temperature, may be consistent with the conformational flexibility expected for a protein whose biological function involves a variety of conformational changes. On the other hand, for enzymes that simply catalyze a chemical reaction, one may expect the denaturation temperature to be above the environmental temperature so that the protein is in a folded, biologically-functional state as often as possible under physiological conditions. However, it is reasonable that the denaturation temperature should only be a few degrees above the environment temperature because: i) once the denaturation temperature value guarantees that most protein molecules are in their native functional state at the physiological temperature there should be no selective pressure for further increases in denaturation temperature; ii) there is plenty of computational and experimental evidences pointing out a universal distribution of mutational effect on protein stability [21, 22]. Moreover, most mutations in a protein are destabilizing [15–25] with very few exceptions, often identified as back-to-the-consensus or back-to-the-ancestor mutations [20, 32–34]. Therefore, drift is not expected to increase denaturation temperature much above the evolutionary threshold determined by function. Despite these seemingly reasonable arguments, the denaturation temperature values for the laboratory resurrections of Precambrian β-lactamases, nucleoside diphosphate kinases and thioredoxins (Fig 1) fail to support this reason, in particular those of thioredoxins which are ~50 degrees above ancient environmental temperatures estimated from the isotopic composition of cherts. This paradox remains, however, even if the interpretation of chert isotopic composition is called into question because the denaturation temperature values for Elongation Factors could be taken minimally as an upper estimate of the environmental temperatures (as is the case for dozens of globular proteins [35]). Furthermore, the ~50 degrees difference also holds when considering modern thioredoxins. Fig 2 is a plot of T_m_ *versus* T_ENV_ that includes data for both, resurrected ancestral thioredoxins and several modern thioredoxins. The ancient and modern proteins conform to a common pattern in this plot and show denaturation temperatures about 50 degrees above the environmental temperatures (estimated ancestral temperatures for the resurrected ancestral thioredoxins and organismal growth temperatures for the modern thioredoxins). Note the enormous time scale implicit in Fig 2: humans and *E*. *coli* share a common ancestor about 4 billion years ago and, therefore, *E*. *coli* and human cytosolic thioredoxins are separated by 8 billion years of evolution (actually, they show only 26% sequence identity). Still, the denaturation temperatures of these two proteins are similar and are ~50 degrees above the environmental temperature (~37°C in both cases).

The fundamental issue underlying the discrepancies described above is our general lack of understanding of the factors that determine the relation between protein denaturation temperatures (T_m_) and the corresponding environmental temperatures of host organisms (the environmental temperature, T_ENV_). A good correlation between T_m_ and T_ENV_ has sometimes been claimed [35] and may likely hold for some set of homologous proteins [1, 10, 27]. However, Dehouck and coworkers have analyzed a dataset of 127 monomeric protein families [36] and have noted that some mesophilic organisms contain proteins with very high denaturation temperatures and that the denaturation temperatures of human proteins in their dataset spanned a range of 39–90°C (for instance, the denaturation temperature for human thioredoxin is about 90°C [11]). The latter results are perplexing given that the environmental temperature for most human proteins is 37°C. In fact, these authors found a weak correlation between T_m_ and T_ENV_ for their 127 family dataset (correlation coefficient 0.59) and, furthermore, they demonstrated that such limited correlation partially reflected the self-evident requirement that T_m_≥T_ENV_ [36].

### Protein stability is not a simple property; it rather reflects a diversity of measures for functionality under different conditions and time scales

Protein denaturation temperatures may be much higher than the temperatures of their host microorganisms because the difference in T_ENV_-T_m_ is dependent upon protein context. Yet, the two temperatures are sometimes correlated when considering a set of homologs from several hosts (see, for instance, Fig 2A in [1] and Fig 1 in [27]) and also when considering laboratory resurrections of Precambrian proteins, as seen by the data in Figs 1 and 2. This curious result (i.e., ΔT_m_≈ΔT_ENV_ even if T_m_>>T_ENV_) is likely related to the notion that protein stability is not a simple property; it rather encompasses a diversity of metrics for the persistence of a functional state under different conditions and time scales. We propose that, in many cases, natural selection for protein stability does not operate on the basis of denaturation temperature, but rather on the basis of other stability-related features that, under certain conditions, correlate with denaturation temperature. This notion is expounded upon below.

Academic literature has traditionally emphasized thermodynamic protein stability (unfolding free energy change and related quantities) as determined from *in vitro* experiments, typically with small “model” proteins, in which reversible, equilibrium, two-state unfolding is observed [37–39]. On the other hand, for technological and biomedical applications of proteins that require stabilized variants, stability is often described in terms of parameters such as the rates of inactivation at the temperature of the application, the shelf life for a protein pharmaceutical, the serum half-life for a therapeutic protein, etc. [40, 41]. These metrics actually describe the time-scale upon which irreversible denaturation takes place by processes such as aggregation, proteolysis, chemical alteration of residues, undesirable interactions, etc. [18, 40–50]. Several molecular features may be responsible for protein kinetic stability, although the most obvious one is a high free energy barrier for unfolding, defined as the difference in free energy between the native state and the kinetically-relevant transition state for unfolding (the state “at the top” of the rate-determining free-energy barrier). The reason is that if irreversible denaturation processes (aggregation, proteolysis, etc.) take place from unfolded or partially-unfolded protein states (the so-called Lumry-Eyring scenario), a high free-energy for unfolding will serve as a safety measure in the sense that the rate of protein irreversible denaturation in an harsh environment will never be faster than the rate of “crossing over” the unfolding free-energy barrier.

### Natural selection for protein stability may operate on the basis of kinetic stability features (such as denaturation rates) that in-turn determine *in vivo* protein lifetime

Our motivation to characterize protein stability stems from the large disparity over a planetary time scale between denaturation temperatures for laboratory resurrections of Precambrian proteins and plausible estimates of environment temperatures (Fig 1). What is important in this context is not, in principle, which metrics academia or industry have deemed convenient to use to describe protein stability, but, rather, which stability-related features are relevant *in vivo* and are subject to natural selection over evolutionary time scales. While there is little doubt that many proteins are naturally selected to be thermodynamically stable (a positive unfolding free energy under physiological conditions), many *in vivo* studies into intracellular protein stability focus on the degradation of individual proteins as followed, for instance, by radiolabelling, pulse-chase experiments or single-cell fluorescence measurements on fusions of the protein of interest with the green fluorescent protein [51–54]. These studies have revealed a remarkably wide span of intracellular stabilities, as shown by *in vivo* half-lives that range from values on the order of seconds to values on the order of years. Obviously, these amply different values are the result of natural selection acting on intracellular stabilities, with, for instance, enzymes involved in regulation showing short *in vivo* half-lives [55] versus proteins involved in exceptionally stable cellular structures that display long *in vivo* half-lives [54]. The molecular determinants of the rates of *in vivo* protein degradation are not well understood. Roles for factors such as molecular weight, accessible surface area, isoelectric point and specific sequence motifs have been proposed and explored, but no clear conclusion has emerged ([52] and references quoted therein). We propose that, since protein degradation *in vivo* occurs fundamentally through proteolysis [56], molecular features that determine resistance to proteolysis *in vitro* may also be relevant for *in vivo* intracellular stability. These features may include in some cases the suppression of local structural fluctuations that render the native state susceptible to proteolysis [43] and, in general, a sufficiently high free-energy barrier for unfolding, since the protein states on the other side of the barrier (unfolded and partially unfolded) are highly vulnerable to proteolytic attack and, therefore, the rate on proteolysis may be determined by the rate of unfolding [45, 57]. Note that if complex machines dedicated to protein degradation, such as the proteasome, can catalyze protein substrate unfolding [58] and bypass the barrier, a protective unfolding free-energy barrier may be needed to ensure kinetic stability before the protein is targeted for degradation. Overall, our model proposes a plausible correlation between *in vivo* stability that is subject to natural selection and protein kinetic stability as assessed from *in vitro* experiments on irreversible protein denaturation [41]. Furthermore, our model suggests a plausible rationalization for differences between protein denaturation temperature and environmental temperature, and may ultimately lead to procedures to estimate the latter from the former.

### Summary of the proposed approach and some general considerations about its applicability to thioredoxins

We propose a simple model of protein stability evolution that includes selection for both thermodynamic stabilization and kinetic stabilization. Our analyses of this model show that selection for kinetic stability (denaturation rate) can naturally lead to protein T_m_ values many degrees higher than the living temperature of a host organism (the environment temperature, T_ENV_). Analyses also reveal plausible scenarios under which variations in host T_ENV_ are mirrored by changes in protein T_m_ (even if T_m_>>T_ENV_). One such scenario involves the following features: i) the *in vivo* protein lifetime is approximately conserved (within a few orders of magnitude); ii) the *in vitro* protein T_m_ values reflect equilibrium protein unfolding; iii) the transition state that determines kinetic stability is substantially unstructured. As we describe in some detail below, this proposed scenario should reasonably apply to thioredoxins.

Previous studies have shown that the in vitro thermal denaturation of *E*. *coli* thioredoxin conforms to a two-state equilibrium model [16, 17, 59] and experimental data reported here support that the same holds to an acceptable extent for ancestral resurrected thioredoxins. In vitro denaturation temperatures for thioredoxins are thus expected to reflect equilibrium protein unfolding (feature ii of the proposed scenario).

Previous experimental analyses on the effect of 27 mutations on the thermal denaturation and unfolding rates of *E*. *coli* thioredoxin [18] revealed an excellent correlation between unfolding activation free energy (the free energy barrier that determines unfolding rate) and the unfolding free energy changes. The correlation was well described by a linear equation of zero intercept and slope close to unity (see figure 8 in ref. 18), supporting that the mutations studied had been introduced in regions that were unstructured in the transition state for unfolding. In fact, the mutations studied were performed at 27 different positions (a substantial number of positions for a protein of 108 residues) scattered throughout the thioredoxin structure (see figure 1 in ref. 18) and included both surface positions and buried positions at the two hydrophobic cores of the thioredoxin molecule. Clearly, the assumption of a substantially unstructured transition state for unfolding (feature iii of the proposed scenario) is certainly reasonable for *E coli* thioredoxin. Furthermore, data reported here on solvent exposure in the transition state support that it should also be a reasonable assumption for the resurrected ancestral thioredoxins studied.

It is important to note at this point that a "substantially unstructured transition state" does not mean that the transition state is fully unfolded. Obviously, some (presumably small) region of the thioredoxin structure must remain structured in the transition state (otherwise the transition state would be the same as the unfolded state, which does not make sense). The important point in the context of this work is that, if the transition state is substantially unstructured, a large number of mutations will show similar effects on equilibrium unfolding free energy and activation free energy. As it will be shown here in detail, this congruence between mutational effects on unfolding equilibrium and unfolding rates will produce a correlation between the equilibrium T_m_ values and the T_ENV_ values and, under selection for kinetic stability, the T_m_ values will track the T_ENV_ values while remaining many degrees above.

The analyses reported here are based upon the assumption that *in vivo* irreversible denaturation of thioredoxin requires complete unfolding (i.e., requires crossing the free energy barrier for complete unfolding), while some proteins can certainly denature upon partial unfolding followed by aggregation or proteolysis. Regarding this possibility, it must be noted that a free-energy congruence between the effect of a given mutation on the unfolding equilibrium and unfolding rates will hold regardless of whether the congruence is linked to complete unfolding or to local unfolding at protein region that includes the position mutated. It follows that, if a diversity of local unfolding processes at many different protein regions can lead to irreversible denaturation, the equilibrium/rate congruence will hold for many mutations throughout the protein structure and the situation would equivalent to that predicted by the complete unfolding scenario. Still, in the case of thioredoxins, it is likely that we do not need to invoke this interpretation because our previous studies do support that proteolysis of *E*. *coli* thioredoxin does require crossing the main free energy barrier for complete unfolding [57]. This result is not surprising because natural selection for kinetic stability may have led, not only to sufficiently high free energy barriers for complete unfolding, but also to suppression or substantial reduction of dynamic fluctuations that render a protein susceptible to proteolysis, as is the case of the α-lytic proteases studied by Agard and coworkers [43].

We conclude, overall, that the scenario described in the first paragraph of this section should plausibly hold for thioredoxins to a substantial and, therefore, we will use this proposed model, together with the experimental denaturation rates for resurrected thioredoxins, to derive estimates of environmental temperatures for Archaean life. Modifications of the proposed model for other protein systems will be dealt with in the Discussion section.

## Results

### Simple models of protein stability evolution that include selection for thermodynamic stabilization and kinetic stabilization

We will consider in this section a protein that denatures *in vitro* reversibly according to a two-state equilibrium model:

N⇄U (1)

The *in vitro* measured denaturation temperature is then that temperature at which the unfolding equilibrium constant is at unity.

$$K=\frac{{\left[U\right]}_{EQ}}{{\left[N\right]}_{EQ}}$$ (2)

In Eq 2, [U]_EQ_ and [N]_EQ_ are the concentrations of the unfolded and the native states at equilibrium (see Materials and Methods for the integrated van`t Hoff equation that gives the temperature dependence of K and, in general, for the detailed mathematical analysis of the proposed model). The equilibrium constant at any given temperature is related to the unfolding free-energy change (the difference between the free energies of the unfolded and native states: ΔG = G_U_-G_N_) through the well-known thermodynamic relationship:

K=exp(−ΔGRT) (3)

Many studies [15, 18, 19, 23, 60–62] support the notion that the main features of the evolution of protein stability can be understood in terms of stability thresholds, i.e., mutations are selected against or rejected when they bring stability below the threshold (purifying selection). Therefore, we consider now the protein in the intracellular milieu (i.e. *in vivo*, rather than *in vitro*) and we explore the nature of the relevant stability thresholds. To this end, we analyze two very simple scenarios corresponding to thermodynamic control of *in vivo* stability and strong kinetic control of *in vivo* stability:

#### Thermodynamic control of *in vivo* protein stability

We assume here that the rates of synthesis and degradation are slow compared with the rates of folding-unfolding and, therefore, the folding-unfolding equilibrium is established *in vivo*. In this very simple model, the balance of synthesis and degradation determines the total protein concentration and the fraction of unfolded protein (non-functional) is determined by the unfolding equilibrium constant (Eq 2). Under this model, mutations would be accepted or rejected according to a stability threshold linked to the value of the unfolding equilibrium constant (K). This result makes sense for sufficiently high values of K, where fitness could be compromised due to the high fraction of non-functional unfolded protein at equilibrium.

We model this K-linked threshold using the following simple fitness function:

fEQ=1 if K≤K* and    fEQ=2(KK*)(KK*)+1 if K>K* (4)

where the fitness function (f_EQ_) equals unity when K is smaller than the threshold value (K*) and decreases sharply when the threshold is violated (K>K*). Our analyses indicate that K* is unlikely to be much smaller than about 10^−2^ because for K equal to 10^−2^, essentially all the protein is in the native state and further decreases in K will barely change the equilibrium amount of functional protein. This, of course, does not mean that the threshold K* cannot be larger than 10^−2^. It only means that it will be difficult to explain a value of K* substantially smaller than 10^−2^ on the basis of natural selection for thermodynamic stability alone. (See “Simple Analysis of Flux under Thermodynamic and Kinetic Control of Enzyme Stability” of S1 File for details.)

#### Strong kinetic control of *in vivo* protein stability

We specifically consider now the kinetics of protein degradation. We assume that degradation occurs mainly through irreversible processes (proteolysis, aggregation, etc.) of the unfolded state, in such a way that denaturation *in vivo* can be represented as a first approximation in terms of a Lumry-Eyring mechanism [41, 42, 63, 64]:

N⇄U→F (5)

where F is the final, irreversible-denatured state (aggregated protein, proteolyzed protein, etc.). Straightforward analysis of this mechanism (see Materials and Methods) leads to the following expression for the degradation half-life (i.e., the *in vivo* life time):

$$\tau =\frac{1}{k_{U}}+\frac{1}{kK}$$ (6)

where k is the rate constant for the irreversible denaturation step (U→F) and k_U_ is the rate constant for the unfolding step (N→U) which is given by transition-state theory as:

$$k_{U}=k_{0}exp\left(-\frac{\Delta G^{‡}}{RT}\right)$$ (7)

where k_0_ is the front factor and ΔG^‡^ is the free energy barrier for unfolding, i.e. the free-energy difference between the native and the transition state (ΔG^‡^ = G^‡^-G_N_), the latter corresponding to the hypothetical structure (or ensemble) at the top of the barrier. Actually, if the irreversible denaturation step is sufficiently fast (large value of k), 1/kK is very low, Eq 6 simplifies to,

$$\tau ≅\frac{1}{k_{U}}$$ (8)

and the degradation half-life is determined by the rate of unfolding. Eq 8 simply recapitulates the well-known result that the kinetic stability of a protein may be determined in large part by the unfolding free-energy barrier. In this scenario, the amount of functional native state is determined entirely by kinetics and, under a very simple model, it is given by [N] = r_S_·τ, where r_S_ is the rate of synthesis. The amount of native protein would then increase with the value of the degradation half-life (i.e., it would increase as the protein becomes more kinetically stable). However, the dependence of metabolic flux (or, simply, the rate of an enzyme-catalyzed reaction *in vivo*) with enzyme activity typically displays a saturation profile [65, 66]. Therefore, while sufficiently low degradation half-lives may compromise fitness, increasing τ beyond the values that essentially reach flux saturation should have little effect. Clearly, we have a threshold-like situation that we describe in terms of the following fitness function:

fDEG=1 if τ≥τ* and    fDEG=2(ττ*)(ττ*)+1 if τ<τ* (9)

which gives fitness values that are equal to unity when the threshold (τ*) is not violated, but that decrease sharply when it is violated. (See “Simple Analysis of Flux under Thermodynamic and Kinetic Control of Enzyme Stability” of S1 File for details.)

It is worth mentioning at this point that, even under the assumption that stability *in vivo* is entirely determined by kinetics, natural selection for thermodynamic stabilization cannot be disregarded. The reason is that, with the obvious exception of intrinsically disordered proteins, the native state is expected to be thermodynamically favored with respect to the unfolded state to guarantee that folding occurs upon protein synthesis (even if, after folding has occurred, the stability of the native state may rely exclusively on kinetics). Accordingly, we pose a fitness function as a product of two terms related to threshold selection for thermodynamic stability and threshold selection for kinetic stability:

$$f=f_{EQ}f_{DEG}$$ (10)

where f_EQ_ and f_DEG_ are given by Eqs 4 and 9, respectively. The product in Eq 10 accounts for the obvious fact that, even with sufficient kinetic stabilization (f_DEG_ = 1) organismal fitness is compromised if folding upon protein synthesis is inefficient because of insufficient stabilization of the native state.

In each step of our simulations, a mutational change in the unfolding free energy (ΔΔG) at a given reference temperature is extracted from a distribution inspired by the results of experimental studies into mutation effects on protein stability (see Materials and Methods for details). As such, in accordance with experimental and computational results [15–25] we use a distribution that predicts mutations to be destabilizing (ΔΔG<0) in most cases and only occasionally stabilizing (ΔΔG>0). The corresponding changes in unfolding free energy barrier are calculated as,

$$\Delta \Delta G^{‡}=\phi \Delta \Delta G$$ (11)

where a transition-state ϕ-value is included. According to the simplest, “classical” interpretation, ϕ~0 for mutations in regions that remain structured and native-like in the transition state while ϕ~1 for mutations in regions that are substantially unstructured or unfolded in the transition state [67, 68]. Note that we are actually using here the definition of ϕ_unf_ as originally introduced by Fersht and coworkers [67]. Once the mutation-induced changes in unfolding free energy and unfolding free-energy barrier are determined, it is possible to calculate the values of the unfolding equilibrium constant, the unfolding rate constant, the degradation half-life and the fitness functions at a given environment temperature, T_ENV_, as well as the denaturation temperature, T_m_ (see Materials and Methods for details). Mutations that increase total fitness (i.e., f in Eq 10) are accepted, while mutations that decrease fitness are rejected. Some simple population genetics models predict low probability of fixation for neutral mutations [69]. In our simulations, mutations that do not change fitness (for instance, mutations that simply keep the equilibrium constant and degradation half-life above the corresponding thresholds and, therefore keep the fitness value at unity) are accepted randomly with a probability of 0.01. Further analysis is discussed in detail in “Some general comments on the proposed model of protein stability evolution” of S1 File.

### Large discrepancies between protein denaturation temperatures and organismal environmental temperatures cannot be explained on the basis of natural selection for thermodynamic stability

**Fig 3:**
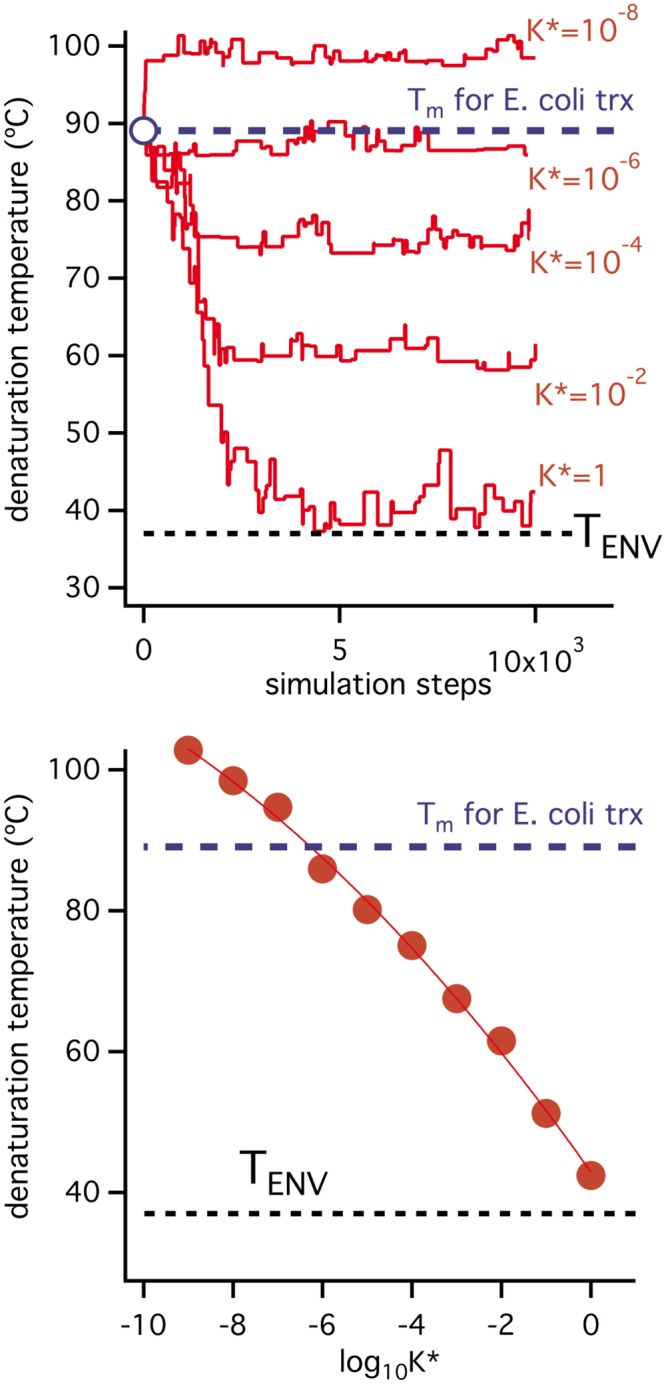
Simulations on the evolution of protein denaturation temperature under selection for thermodynamic stability only. All simulations assume an environmental temperature of 37°C and use as starting denaturation parameters those corresponding to *E*. *coli* thioredoxin (including a denaturation temperature value of 89°C which is about 50 degrees higher than the assumed environmental temperature). Upper panel: simulations performed using different values for the threshold value of the unfolding equilibrium constant. Lower panel: final values of the denaturation temperature obtained in the simulations shown in the upper panel versus the threshold K* values used.

We first explore whether a difference of about 50 degrees between protein denaturation temperature and host environmental temperature (as suggested by the thioredoxin data in Figs 1 and 2) can be explained exclusively in terms of natural selection for thermodynamic stability. The simulations shown in Fig 3 (see Materials and Methods for details) start with the parameters of the wild-type form of *E*. *coli* thioredoxin, including an experimental value of its denaturation temperature, 89°C, which is about 50 degrees higher than 37°C (i.e., the typical environmental temperature for *E*. *coli* and also the environmental temperature value used in these simulations). τ* = 0 is assumed in all the simulations in Fig 3 and, therefore, no selection for degradation half-life (i.e., for kinetic stability) is included. On the other hand, several thresholds for the unfolding equilibrium constant at the T_ENV_ temperature have been used, implying different levels of selection for thermodynamic stability. A very simple analysis of the balance between production, folding-unfolding and degradation (see “Simple analysis of flux under thermodynamic and kinetic control of enzyme stability” of S1 File) supports that a value for the unfolding equilibrium constant of (very roughly) 0.01 (meaning that about 1% of unfolded protein in equilibrium with the native state can be tolerated) is a reasonable lower limit for the K threshold under selection for thermodynamic stability. However, K* = 0.01 brings the denaturation temperature to about 60 degrees, i.e. only about 20 degrees above the host environmental temperature. In fact, the simulations show that, in order to keep the denaturation temperature close to 89°C, an equilibrium threshold of K*~10^−6^ would be required, meaning that only about one unfolded molecule in equilibrium with a million native molecules can be “tolerated” or that a decrease in functional protein concentration of 0.0001% is detrimental. The results shown in Fig 3 support, therefore, that a difference of ~50 degrees between denaturation temperature and environmental temperature can hardly be explained in terms of natural selection for thermodynamic stability. Further analysis is discussed in details in “Analysis of all plausible scenarios that could “save” the interpretation based on natural selection for thermodynamic stability” of S1 File.

### Large discrepancies between protein denaturation temperatures and host environmental temperatures can be explained on the basis of natural selection for kinetic stability

**Fig 4:**
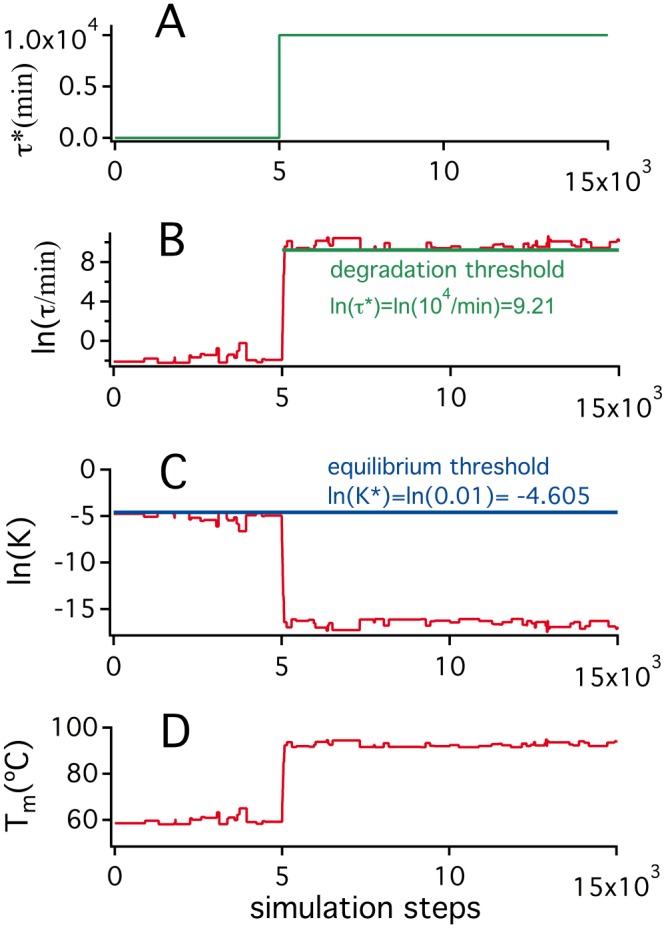
Illustrative example of a simulation on the evolution of protein denaturation temperature under threshold selection for both thermodynamic stability and kinetic stability. The simulation shown assumes an environmental temperature of 37°C and uses as starting state the final state of the K* = 10^−2^ and τ* = 0 simulation in the upper panel of Fig 3. (A) τ* is kept at a zero value for 5000 simulation steps and then it is increased to impose a threshold for degradation half-life of 10^4^ min. (B) Upon τ* increase to 10^4^ min, mutations that decrease the unfolding rate quickly accumulate to bring the degradation half-life above the threshold. (C) The mutations that decrease the unfolding rate, also decrease the unfolding equilibrium constant which, as a result, falls order of magnitude low the (assumed constant in this simulation) K* threshold; (D) The decrease in unfolding equilibrium constant is reflected in an increase in equilibrium denaturation temperature, as expected from the van’t Hoff equation and the endothermic character of protein unfolding. This increase in denaturation temperature reflects exclusively the threshold for kinetic stabilization, as K remains orders of magnitude below K* once τ* = 10^4^ min has been imposed (therefore, changing K* within reasonable limits would not affect the denaturation temperature value).

**Fig 5:**
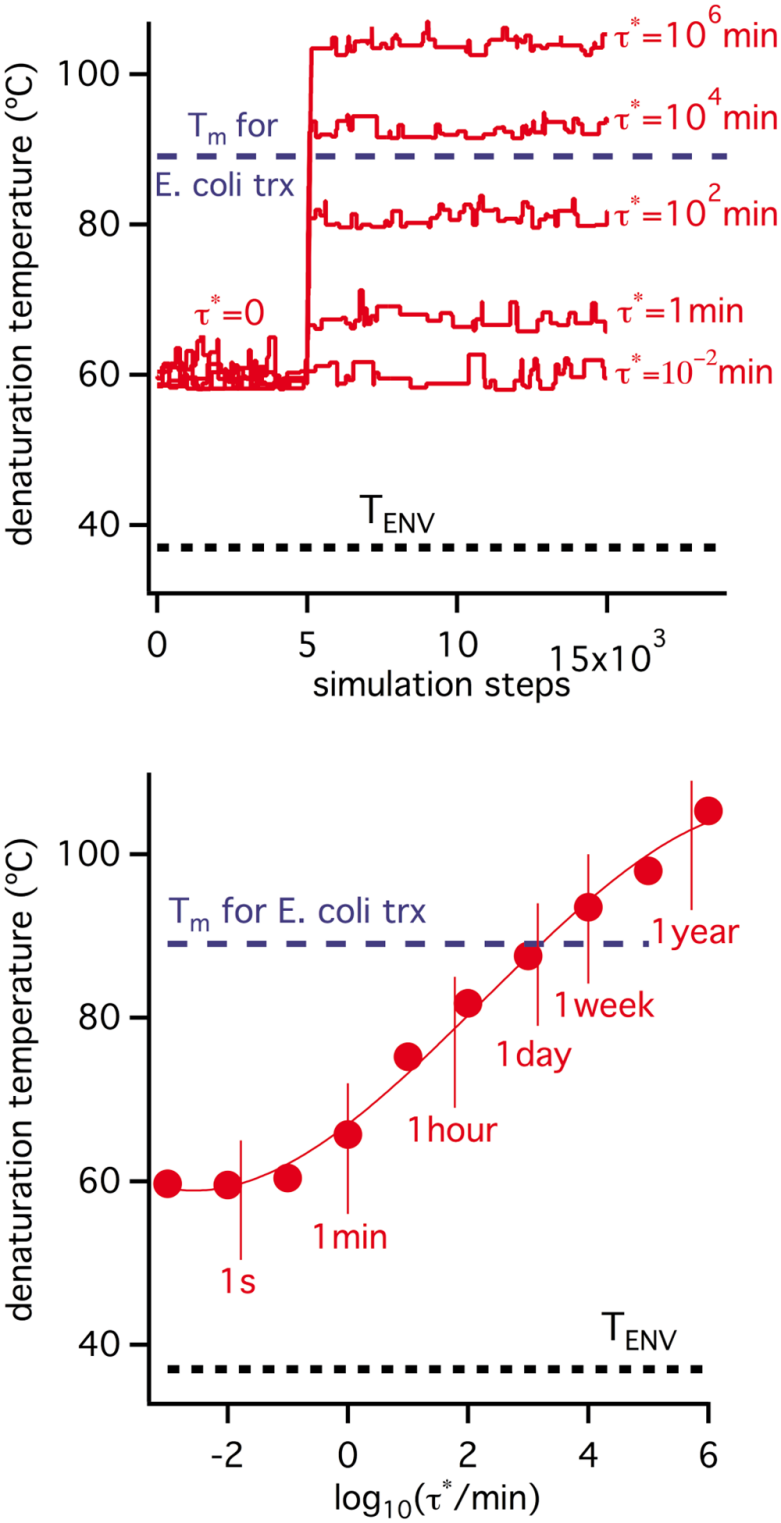
Simulations on the evolution of protein denaturation temperature under threshold selection for both thermodynamic stability and kinetic stability. All simulations assume an environmental temperature of 37°C and use as starting state the final state of the K* = 10^−2^ and τ* = 0 simulation in the upper panel of Fig 3. τ* is kept at a zero value for 5000 simulation steps and then it is increased to impose different levels of selection for kinetic stability. Upper panel: simulations performed using different threshold values for the degradation half-life (τ*). Lower panel: final values of the denaturation temperature obtained in the simulations shown in the upper panel versus the threshold τ* used.

We conclude from the simulations shown in Fig 3 and the discussions above that the T_m_ vs. T_ENV_ discrepancy for *E*. *coli* thioredoxin can hardly be explained in terms of selection for thermodynamic stability. The simulations we include in Fig 3 are intended to suggest that the discrepancy can, on the other hand, be reasonably explained in terms of selection for a suitable degradation half-life (i.e., selection for kinetic stability). All simulations in Figs 4 and 5 start from the final state of the K* = 0.01/τ* = 0 simulation of Fig 3, in which a denaturation temperature of about 60°C is reached. However, a τ* threshold is imposed after a given number of steps, thus generating an evolutionary pressure to increase the degradation half-life, τ. Fig 4 reports the details of the specific simulation in which τ* = 10^4^ min is imposed and a sudden increase in degradation half-life is observed. This increase is achieved through mutations that enhance the activation free energy barrier (ΔG^‡^, Eq 7) and, consequently, decrease the unfolding rate (see Eqs 7 and 8). However, since we are assuming a substantially unstructured transition state, at least at the mutational sites (ϕ = 1 in Eq 11), the mutations that increase ΔG^‡^ are those that increase the unfolding free energy change, ΔG, the unfolding equilibrium constant (Eq 3) and, ultimately, the equilibrium denaturation temperature, T_m_ (temperature at which K = 1). Accordingly, the simulation of Fig 4 shows a large increment in T_m_ value upon implementation of the τ* threshold. It is important to note that this increase in T_m_ value reflects exclusively the kinetic stability (degradation) threshold. The reason is that, upon imposition of τ* = 10^4^ min, the unfolding equilibrium constant decreases for the reasons described above and becomes orders of magnitude lower than the K* threshold (panel C in Fig 4, see also S1 and S2 Figs for a clear illustration). Therefore, there is no risk of equilibrium threshold violation with τ* = 10^4^ min and, in fact, the result of the simulation is quite insensitive to changes in K* within reasonable limits. The implication is that in the simulation of Fig 4, as well as in all subsequent simulations reported, the resulting T_m_ values are well determined in the sense that they cannot be obtained from several different combinations of K* and τ* thresholds. Loosely speaking, the T_m_ values obtained from the simulations reported here are not contaminated by “overfitting”.

Fig 5 shows the results of several simulations identical to that in Fig 4 except for the τ* threshold used. Denaturation temperatures increase with the τ* value and, in fact, a denaturation temperature about 50 degrees above the host’s environmental temperature (T_ENV_ = 37°C) is obtained with a half-life threshold on the order of days. Although we have not been able to find in the published literature an experimental value for the *in vivo* lifetime of *E*. *coli* thioredoxin, a lifetime of a few days or so seems entirely plausible, as many proteins show lifetimes in this range [51–54] and, in fact, for some “thioredoxin-like” proteins, *in vivo* lifetimes of about one day have been reported [52] and a somewhat smaller lifetime (~7 hours) has been reported for a thioredoxin in human cancer cells [70].

### Natural selection for kinetic stability may be consistent with equilibrium denaturation temperatures correlating with organismal environmental temperature, while remaining substantially higher

**Fig 6:**
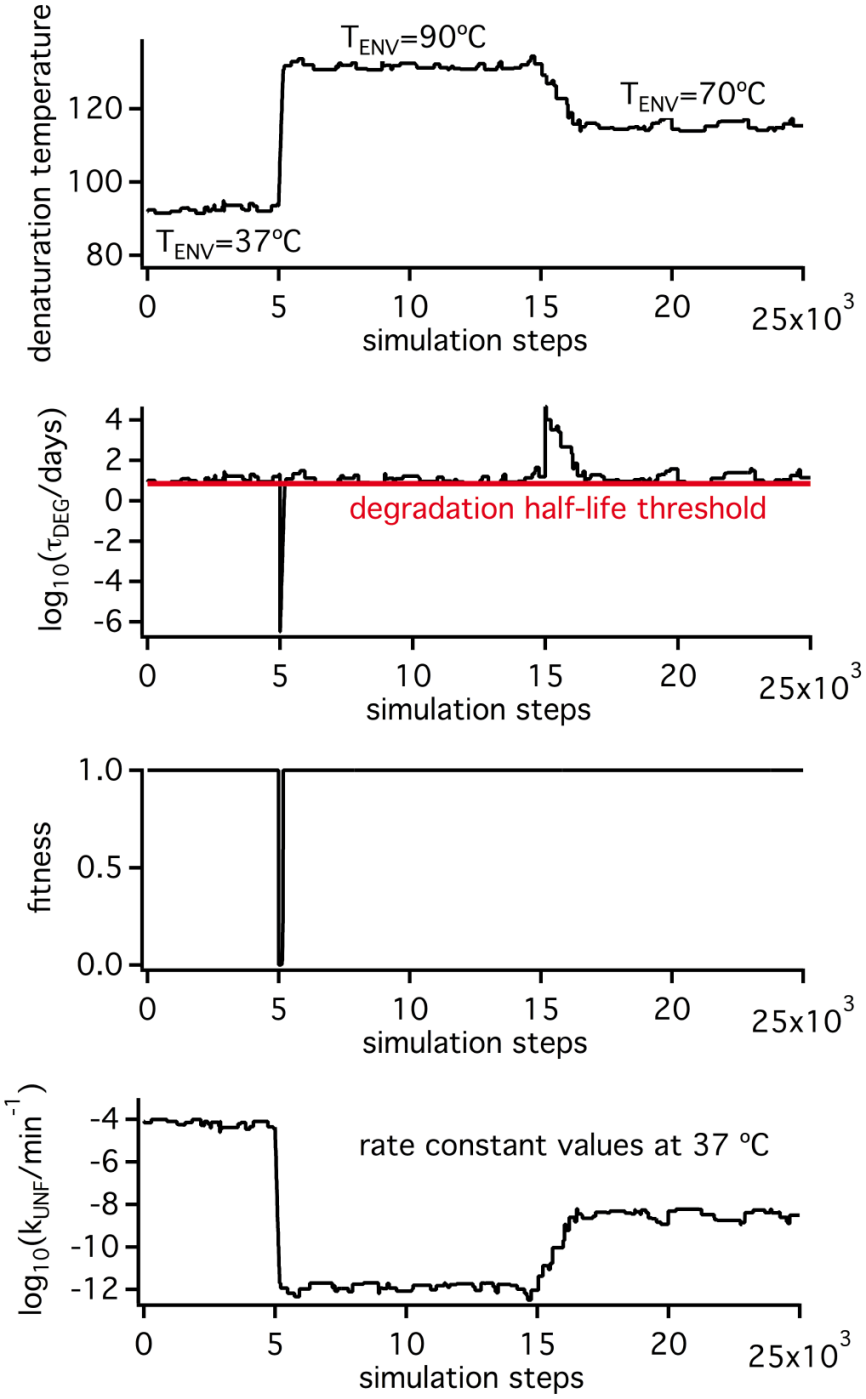
Simulations on the response of protein denaturation temperature to changes in environmental temperatures under conditions of threshold selection for kinetic stability. The simulation starts with the final state of the T_ENV_ = 37°C and τ* = 10^4^ min of Fig 5. However, after 5000 simulation steps, the T_ENV_ value is increased to 90°C and, after 15000 simulation steps, it is decreased to 70°C. Profiles of denaturation temperature, degradation half-life, organismal fitness and unfolding rate constant are shown.

**Fig 7:**
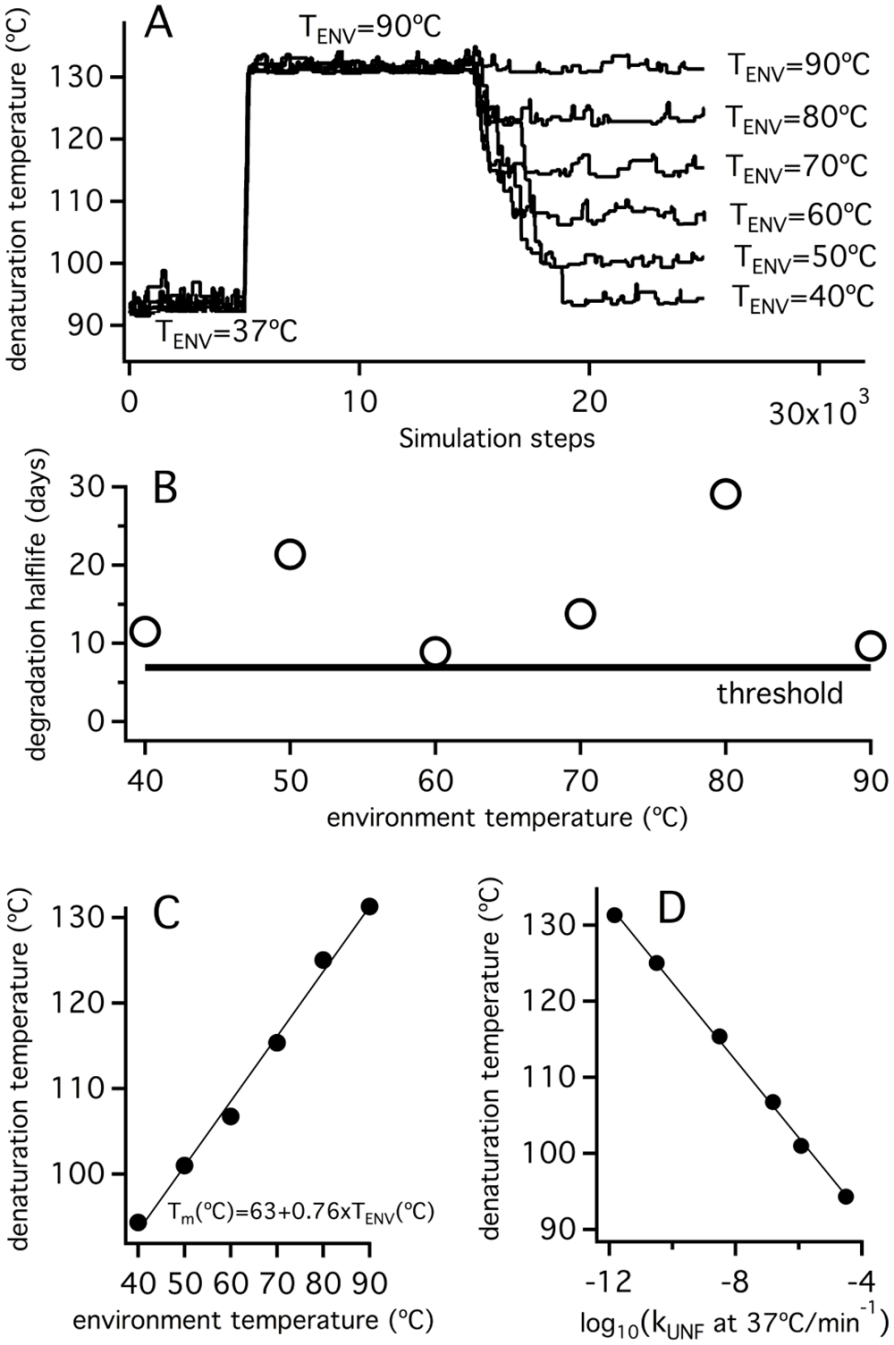
Simulations on the response of protein denaturation temperature to changes in environmental temperatures under conditions of threshold selection for kinetic stability. The simulations shown are identical to that displayed in Fig 4, except that different values for the final environmental temperature are used (panel A). Plot of the final values for the degradation half-life (B) and the denaturation temperature (C) versus environment temperature are displayed, together with the correlation between the final values of the denaturation temperature and the logarithms of the unfolding rate constants at 37°C (D).

The simulation shown in Fig 6 explores the response of the denaturation temperature value to changes in environmental temperature once selection for kinetic stability dominates. The simulation starts with the final state of the τ* = 10^4^ min and T_ENV_ = 37°C simulation from Fig 4. However, after a given number of steps, the environmental temperature is increased to 90°C, kept at that value for 10000 steps and finally decreased to 70°C. The initial increase to T_ENV_ = 90°C brings about an immediate violation of the degradation threshold (see the fitness profile in Fig 6) and triggers the accumulation of stabilizing mutations to decrease the rate of unfolding. Of course, these mutations also increase the unfolding equilibrium constant and, as a kind of “side effect”, increase the value of the denaturation temperature. The second change in environmental temperature from 90°C to 70°C decreases the unfolding rate, increases the degradation half-life and, therefore, does not bring about a threshold violation. Yet, since most mutations are destabilizing, random drift increases the unfolding rate and brings the degradation half-life to a value close to the threshold. Obviously, this neutral accumulation of destabilizing mutations affects the denaturation temperature, which eventually reaches a value consistent with (but higher than) a T_ENV_ of 70°C. Simulations in Fig 7 are replicas of that in Fig 6, except that different final environmental temperatures are used. It becomes apparent that the denaturation temperature value reached at the end of each simulation tracks with the T_ENV_ value used and, in fact, a plot of T_m_ versus T_ENV_ reveals an excellent linear correlation with a slope close to unity, although somewhat smaller (panel C in Fig 7). Still, the T_m_ values are about 50 degrees higher than the corresponding T_ENV_ values, suggesting that the outcome of the simulation is determined by the imposed selection for kinetic stability and that the T_m_ values simply reflect changes in kinetic stability (see also S2 Fig).

### *In vitro* experimental signatures of the proposed selection-for-kinetic-stability scenario

Overall, the simulations reported here reveal a plausible scenario under which equilibrium denaturation temperatures correlate with an organism’s growth temperature, while remaining many degrees higher. Such a scenario involves several key features: 1) natural selection for kinetic stability operates; 2) the associated threshold level for the *in vivo* protein lifetime remains reasonably constant across several organisms under consideration, even if those organisms grow at different environmental temperatures; 3) the transition state that determines the rate of irreversible denaturation *in vivo* is substantially unstructured and, therefore, mutations that enhance kinetic stability also enhance the unfolding free energy, the unfolding equilibrium constant and the equilibrium denaturation temperature.

The simulations reported suggest that the above scenario may be reflected in a number of *in vitro* experimental signatures: 1) *in vitro* denaturation temperature corresponds to an equilibrium unfolding process; 2) the half-life times for *in vitro* unfolding (likely related to the *in vivo* half-life times) at organismal host temperatures are approximately constant (this signature is born out of our simulations: see panel B in Fig 7); 3) there is a good correlation between denaturation temperature and the unfolding rates at a given temperature, indicating that changes in denaturation temperature reflect changes in kinetic stability (again this signature is born out of our simulations: see panel D in Fig 7); and 4) experimental parameters such as chemical-denaturant activation m-values, activation heat capacities indicate that the kinetically-relevant transition state for unfolding is substantially unstructured. We next attempted to determine whether laboratory resurrections of ancestral thioredoxins meet these signatures.

### On the applicability of the two-state equilibrium unfolding model to the *in vitro* thermal denaturation of laboratory resurrections of Precambrian thioredoxins

The computational analyses described above and the subsequent interpretation of the differences between protein denaturation temperature and host environmental temperature rely, among other assumptions, on the *in vitro* denaturation process being described, at least to an acceptable degree of approximation, by the two-state equilibrium model (Eq 1). This is not a trivial point, not only because equilibrium thermal unfolding of several protein systems has been shown to involve significantly populated intermediate states [71, 72], but mainly because, more often than not, *in vitro* protein thermal denaturation is an irreversible rate-limited process that must be described in terms of kinetic models ([41] and references therein). Certainly, there can be little doubt that the *in vitro* thermal denaturation of *E*. *coli* thioredoxin conforms acceptably to the two-state equilibrium model, as several experimental studies have clearly supported [16, 17, 59]. However, the same may not hold for the laboratory resurrections of Precambrian thioredoxins since their experimental denaturation temperatures are very high and, as it has been previously described [73], many processes of irreversible protein denaturation (in particular, chemical alteration of residues) become very fast at temperatures around 100°C and above. We have therefore carried out a detailed differential scanning calorimetry analysis into the thermal denaturation of the laboratory-resurrected thioredoxin with the highest denaturation temperature value (LPBCA thioredoxin with a T_m_ value above 120°C: see Fig 1) in order to assess the extent of the kinetic distortion caused by *in vitro* irreversible processes.

**Fig 8:**
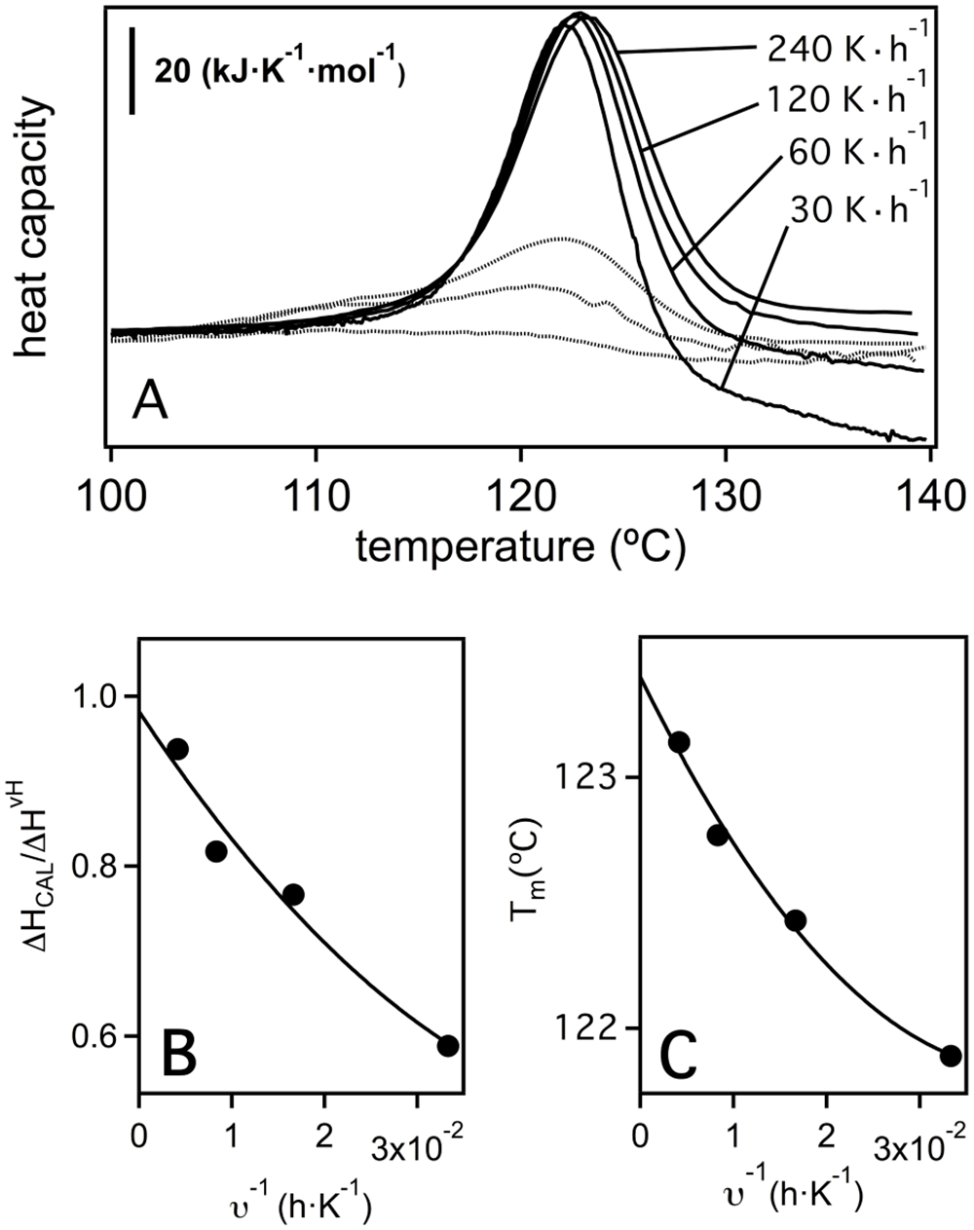
Thermal denaturation of the laboratory representation of the thioredoxin corresponding to the LPBCA node (see Fig 1) as determined by differential scanning calorimetry (DSC). (A) DSC profiles for LPBCA denaturation at several scan rates. Dotted lines represent the profiles obtained in the reheating runs of the experiments at 240, 120 and 60 degrees per hour. (B) Plot of calorimetric to van’t Hoff enthalpy ratio versus one over scan rate. (C) Plot of denaturation temperature (defined here as that corresponding to the maximum of the heat capacity profile) versus one over scan rate. The lines in panels B and C are the best fits of second-order polynomials and are used to extrapolate to infinite scan rate (1/v = 0).

Fig 8 shows DSC calorimetric profiles for LPBCA thioredoxin obtained at different scan rates. Reversibility of the thermal denaturation is tested by performing a second, reheating run after completion of the first one. It is noteworthy that significant reversibility is observed at the higher scan rates employed, despite the fact that the calorimetric runs end at the very high temperature of 140°C. Nevertheless, the degree of reversibility decreases with decreasing scan rate, which is the expected result for a protein thermal unfolding that is subject to partial kinetic distortion: note that the time required for the system to go through the unfolding transition is longer for the slower scan rates, thus allowing the kinetically-determined irreversible alterations to proceed to a larger extent. It is also clear from Fig 8 that the kinetic distortions are mainly confined to the high-temperature side of the calorimetric transitions, where the scan-rate dependence is more apparent.

Theoretical analyses [42, 64] indicate that, for DSC transitions that are subject to partial kinetic distortion because of the occurrence of irreversible alterations, the true (non-distorted) unfolding equilibrium parameters are obtained through extrapolation to infinite scan-rate or, in practical terms, to 1/scan-rate = 0. We have then fitted the experimental DSC transitions for LPBCA thioredoxin with a pseudo-two-state model and we have proceeded to extrapolate to 1/scan-rate = 0, the parameters derived from the fittings. A pseudo-two-state model includes fitting two different unfolding enthalpy values: the calorimetric enthalpy (which determines the area under the calorimetric transition) and the so-called van`t Hoff enthalpy (which determines the temperature-dependence of the unfolding equilibrium constant). For a true equilibrium two-state unfolding, the same value for the two enthalpies values should be obtained and the van`t Hoff to calorimetric enthalpy ratio (r) should equal unity. The values of the ratio r derived from the fits of the pseudo-two-state model to the calorimetric transitions for LPBCA thioredoxin are indeed different from unity, but they approach unity upon extrapolation to 1/scan-rate = 0 (Fig 8). This result suggests that the thermal denaturation of LPBCA thioredoxin can be adequately described as two-state unfolding process that is somewhat distorted by kinetically-determined irreversible alteration processes taking place at high temperature. The distortion introduced by these kinetic processes in the denaturation temperature value is, nevertheless, rather small as shown by the plot of T_m_ versus 1/scan-rate of Fig 8. The estimate of the true value for the equilibrium denaturation temperature obtained from the extrapolation to 1/scan-rate = 0 is actually only about 1–2 degrees higher than the values obtained at the lower scan rates. Such a difference is almost negligible when compared with the range of denaturation temperatures spanned by the extant and laboratory resurrected thioredoxins shown in Fig 1. We take this result as evidence that, for the purpose of the analyses reported in this work, the experimental T_m_ values for the resurrected ancestral thioredoxins can be taken as acceptable estimates of the corresponding equilibrium denaturation temperatures.

### Experimental determination of temperature-dependent unfolding rates for the laboratory resurrections of Precambrian thioredoxins

**Fig 9:**
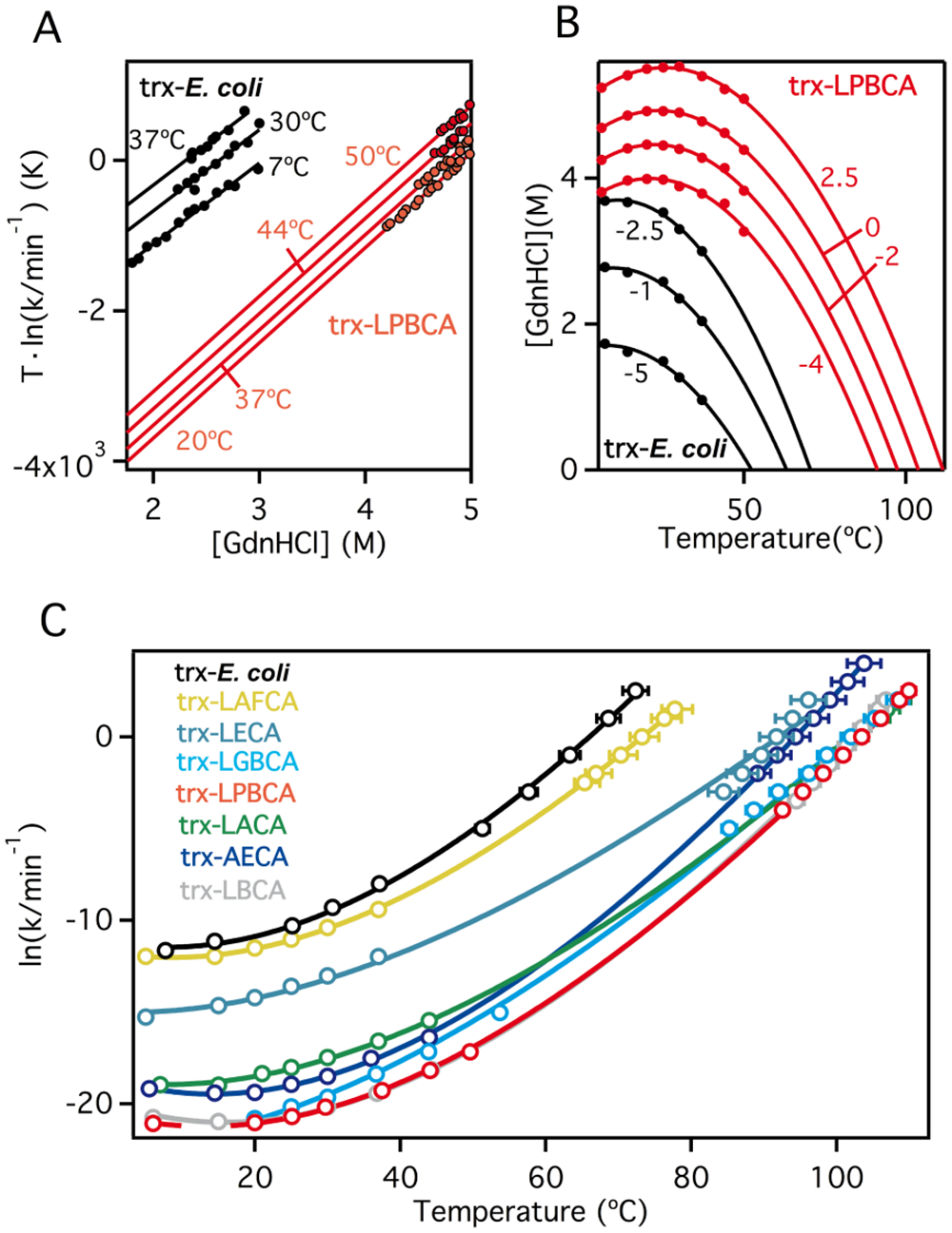
Experimental determination of the temperature-dependent unfolding rate constants for *E*. *coli* thioredoxin and several laboratory resurrections of Precambrian thioredoxins (Fig 1). Guanidine-induced denaturation experiments were performed at several temperatures and guanidine concentration for each thioredoxin variant. Values of the unfolding rate constants (k_U_) were derived from the fits of exponential functions to the corresponding fluorescence profiles. (A) Linear extrapolation to zero guanidine concentration to obtain the values of rate constants at lower temperatures. (B) Constant-ΔG extrapolation to obtain the values of the rate constants at higher temperatures. For illustration, we have included in panels A and B data for *E*. *coli* thioredoxin and LBCA thioredoxin. Data for the other thioredoxins studied are included in the Supporting Information. (C) Profiles of unfolding rate constants (values obtained using the extrapolation procedures illustrated in panels A and B) versus temperature for all the thioredoxins studied in this work. Continuous lines are the best fits of Eq 13 to the data.

*In vitro* signatures of our proposed scenario to explain the correlation between widely different values of T_m_ and T_ENV_ are related with the unfolding rates of the protein variants involved. We have, therefore, carried out an exhaustive experimental study of these rates, including all our laboratory resurrections of Precambrian thioredoxins (Fig 1). For each thioredoxin, the kinetics of chemically-induced unfolding was followed by fluorescence measurements at several temperatures and, for each temperature, at several guanidinium hydrochloride concentrations (see Materials and Methods for details). Unfolding rate constants were determined from the fits of first-order equation to the experimental profiles of fluorescence intensity versus time (see S3 Fig for representative examples) and subsequently extrapolated to zero guanidine concentration in order to obtain the lnk_U_ versus T profile in the absence of chemical denaturant. In fact, the extrapolation was carried out using two different procedures. Following the majority of published literature on the subject, the well-known and commonly used linear extrapolation procedure was employed:

$$lnk_{U}\left(C\right)=lnk_{U}+\frac{m_{u}^{‡}}{RT}C$$ (12)

where k_U_(C) is the unfolding rate constant at the guanidine concentration C, k_U_ is the linear-extrapolation value corresponding to C = 0 and m_u_^‡^ is the activation-m value (a measure of the effect of the concentration of chemical denaturant on the free-energy barrier for unfolding). Global fits of Eq 12 imposing a common m^‡^ value were performed (see Fig 9 and S4–S11 Figs) to minimize the errors associated to a rather long extrapolation. In addition to linear extrapolation, a modification of the constant-ΔG extrapolation we previously introduced [74] was employed. Here, the fits based on Eq 12 are used to find C/T couples corresponding to given values of the unfolding rate constant. Each profile of C versus T for a given k_U_ value can be fit to a second order polynomial and extrapolated to C = 0 to yield the temperature at which the value of k_U_ is obtained in the absence of denaturant (see Fig 9 and S4–S11 Figs).

The advantage of simultaneously using these two extrapolation procedures is twofold. First, the two procedures cover different temperature ranges (see Fig 9 and S4–S11 Figs) and allow for a more precise description of the temperature dependence of the k_U_ values. Second, while the extrapolation based on Eq 12 involves the assumption of a linear concentration dependence of the free-energy change, the “constant-k_U_” extrapolation does not. The fact that the two sets of extrapolated k_U_ values appear consistent with each other (i.e., their temperature dependence can be reasonable described by a single function: see further below) supports that deviations from the linear dependence specified by Eq 12 outside the experimental range are not large. This point is noteworthy because substantial deviations from the linear dependence of unfolding free energies with guanidine concentration have been found in some cases and linked to the efficient screening of surface charge-charge interactions by guanidine concentrations in the approximate 0-1M range [75]. Therefore, the results shown in Fig 9 suggest that the contribution of such interactions is comparatively minor for the thioredoxins studied here.

The profiles of lnk_U_ (extrapolated to zero guanidine concentration) versus temperature where fit with the following integrated Eyring equation:

$$lnk_{U}\left(T\right)=lnk_{U}\left(T_{0}\right)-\frac{\Delta H^{‡}\left(T_{0}\right)}{R}\left\{\frac{1}{T}-\frac{1}{T_{0}}\right\}+\frac{\Delta C_{P}^{‡}}{R}\left\{ln\left(\frac{T}{T_{0}}\right)-\left(\frac{T-T_{0}}{T}\right)\right\}$$ (13)

where T_0_ is a reference temperature (taken as 37°C), ΔH^‡^(T_0_) is the activation enthalpy at the reference temperature and ΔC_P_^‡^ is the activation heat capacity change. Fits of Eq 13 to the experimental profiles were visually confirmed (Fig 9) and the parameters derived from them are collected in Table 1.

**Table 1:** Kinetic parameters for the *in vitro* denaturation of *E*. *coli* thioredoxin and several resurrected Precambrian thioredoxins.

	ln(k_U_/min^-1^)	Δ*H*^*≠*^(*T*_0_)^a^	^$\Delta C_{P}^{\neq }$^	$m_{U}^{\neq }$^b^	ΔASA^c^	^mU≠mEQ^^d^	ΔCP≠ΔCP,EQ^d^
	(*T*_0_)^a^	(kJ·mol^-1^)	(kJ·K^-1^·mol^-1^)^a^	(kJ·mol^-1^·M^-1^)	(Å^2^)		
***E*. *coli***	-8.2 ± 0.1	166.9 ± 2.5	5.8 ± 0.3	8.94 ± 0.12	12108	0.70	~1
**LAFCA**	-9.3 ± 0.0	142.0 ± 0.5	5.01 ± 0.05	8.45 ± 0.14	12372	0.66	0.86
**LECA**	-12.2 ± 0.3	119.0 ± 7.2	3.46 ± 0.45	7.57 ±0.11	12363	0.59	0.60
**LGPCA**	-18.3 ± 0.1	151.3 ± 3.6	4.00 ± 0.17	8.13 ± 0.05	12071	0.64	0.69
**LPBCA**	-19.3 ± 0.1	122.2 ± 1.2	5.14 ± 0.05	10.53 ± 0.10	12467	0.83	0.89
**LACA**	-16.6 ± 0.1	119.3 ± 1.8	4.08 ± 0.08	9.26 ± 0.11	12492	0.73	0.70
**AECA**	-17.5 ± 0.1	132.1 ± 1.2	5.78 ± 0.06	10.53 ± 0.12	12397	0.83	~1
**LBCA**	-19.4 ± 0.1	116.9 ± 2.2	5.53 ± 0.10	10.73 ± 0.10	12273	0.84	0.95

^a^ Activation parameters determined from the fitting of Eq 23 to the rate constant vs. temperature profiles (Fig 9C). Associated errors were determined by bootstrapping.

^b^ Activation m values for the guanidine-induced denaturation of thioredoxins derived from the linear extrapolation fits (Fig 9A).

^c^ Unfolding changes in accessible surface area calculated as ΔASA = ASA(U)-ASA(N). See "Accessibility to solvent calculations" in Materials and Methods

^d^ Estimates of the accessibility to solvent in the transition state for thioredoxins denaturation. See "Accessibility to solvent calculations" in Materials and Methods

### The transition state that determines the kinetic stability of laboratory resurrections of Precambrian thioredoxins is substantially unstructured

Selection for kinetic stability is most efficiently reflected in the equilibrium denaturation temperature value if the kinetically-relevant transition state is substantially unstructured (unfolded) in such a way that mutations that act upon the unfolding free-energy barrier will analogously affect the unfolding free energy change (i.e., the ϕ~1 case in Eq 11). Our exhaustive experimental analysis of the unfolding kinetics for the laboratory resurrections of Precambrian thioredoxins (Fig 9 and Table 1) provides ample information to assess the exposure to solvent in the transition state and, ultimately, the extent to which the transition state is unstructured (since a large exposure to solvent would imply extensive disruption of the native structure).

Chemical-denaturant m values (representing the denaturant concentration dependence of free energy changes: Eq 12 and Fig 9A) are widely accepted to provide a measure of the corresponding changes in accessibility to solvent [76]. The accessibility to solvent of the transition state can then be estimated by comparing the activation m^‡^ value (related to the accessibility difference between the transition state and the native state) with the equilibrium unfolding m_EQ_ value (related to the accessibility change upon complete unfolding). For the thioredoxins studied in this work, the values of the ratio m^‡^/m_EQ_ (Table 1) range between ~0.6 and ~0.8, supporting a transition state with large exposure to the solvent and, therefore, substantially unstructured. An analogous analysis can be performed on the basis of heat capacity changes, which are also known to reflect changes in solvent-accessible surface area [71, 72, 76]. Activation heat capacities (ΔC_P_^‡^ values: Table 1) are determined from the temperature-dependence of the unfolding rate constants (Eq 13 and Fig 9C) and their comparison with the equilibrium unfolding ΔC_P,EQ_ value (related to the accessibility change upon complete unfolding) provides an additional (and independent) estimate of the accessibility to solvent in the transition state. The ΔC_P_^‡^/ ΔC_P,EQ_ ratios range in fact between ~0.6 and ~1 (Table 1), supporting again a substantially unfolded transition state.

A substantially unstructured transition state for thioredoxin unfolding is certainly consistent with our previous mutational studies [18]. It is important, however, to note that we are not claiming that the transition state is "fully unfolded". Rather, we assume that the structured region in the transition state is comparatively small. We discuss this issue in some detail in “Discussions on some approximations used in our simulations of protein stability evolution” of S1 File.

### Estimating ancestral environmental temperatures from the unfolding rates of laboratory resurrected Precambrian thioredoxins

**Fig 10:**
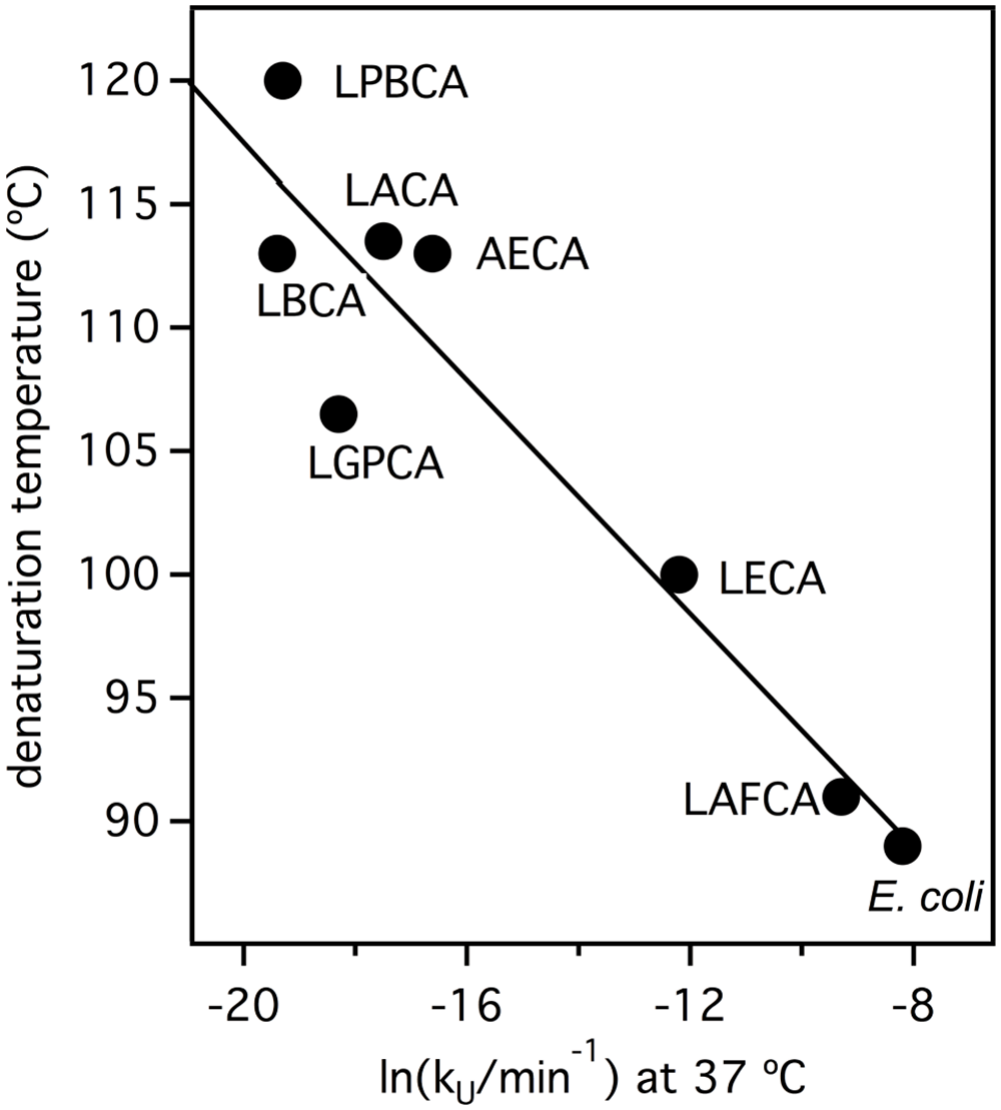
Correlation between the denaturation temperature and the logarithm of the unfolding rate constant at 37°C for all thioredoxins from our current and previous [18] work.

**Fig 11:**
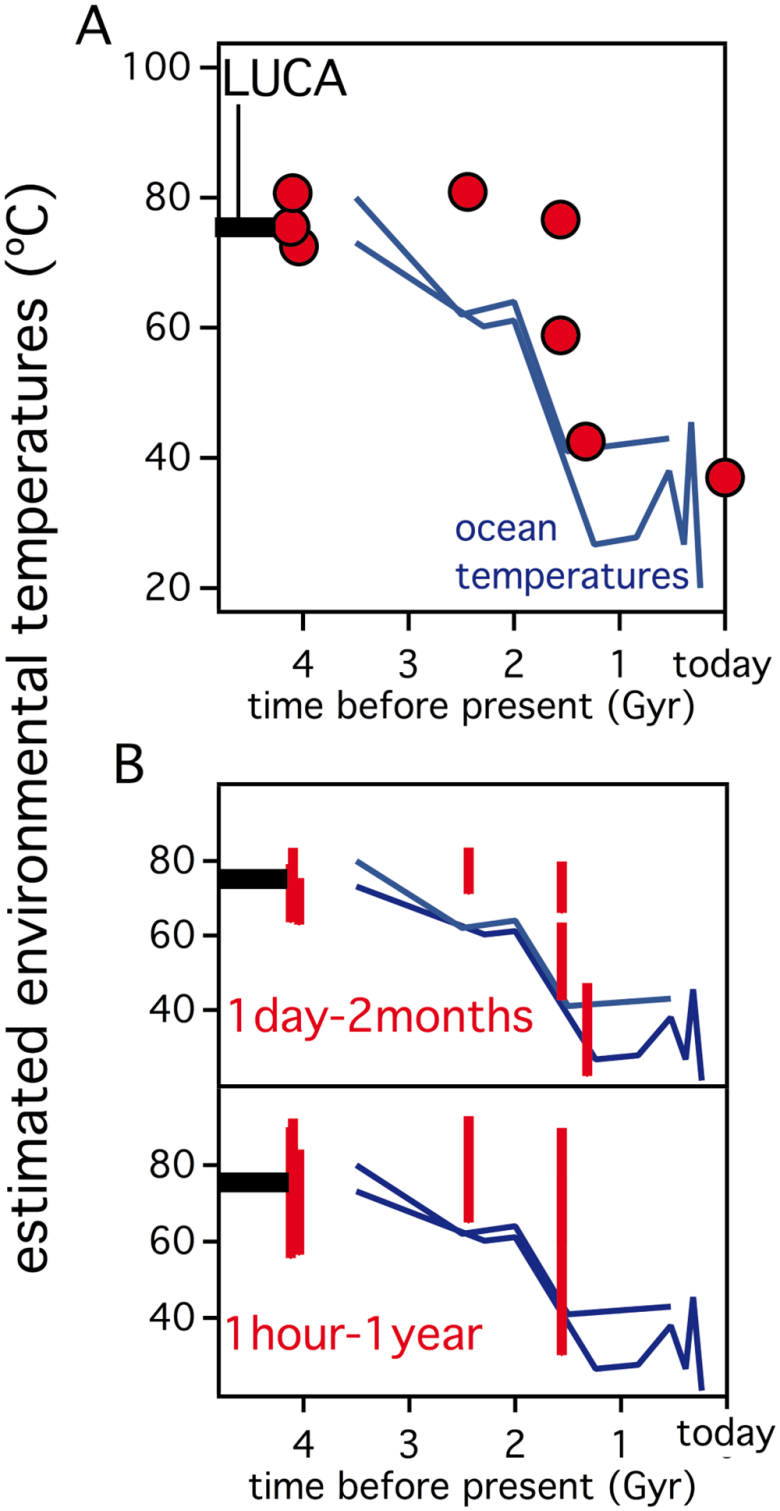
Estimating ancestral environment temperatures from the unfolding rates of laboratory resurrected Precambrian thioredoxins. Environment temperatures are estimated from the unfolding rate profiles of Fig 9C by assuming specific values for the denaturation half-life (τ = 1/k_U_). (A) A τ value (3.8 days) equal to the denaturation half-life of *E*. *coli* thioredoxin is assumed. (B) A one day—two month interval is assumed for τ and the corresponding intervals for the calculated environmental temperature are shown as vertical lines. (C) A one hour—one year interval is assumed for τ and the corresponding intervals for the calculated environmental temperature are shown as vertical lines. In the three panels, the denaturation temperatures for elongation factors and thioredoxins are included, as well as the known estimates of the environmental temperatures derived from the oxygen isotopic composition of cherts. The dashed horizontal line at about 4 billion years (labeled “LUCA”) represents a recent minimum estimate (~75°C) for the environment temperature of the last universal common ancestor [1].

Fig 10 shows that the experimental data for *E*. *coli* thioredoxin and the laboratory resurrections of Precambrian thioredoxins define an excellent correlation between denaturation temperature and the logarithm of unfolding rate at 37°C. As inferred from the simulations described earlier (Fig 7D), this correlation is consistent with the notion that changes in denaturation temperature are a side-effect of natural selection acting upon kinetic stability at a variety of environment temperatures. The possibility arises, therefore, of generating estimates of ancestral environmental temperatures from the *in vitro* unfolding rates, provided that some reasonable assumptions can be made about the ancestral protein lifetimes. The simplest such calculation is shown in Fig 11A. Since we know the environment temperature for *E*. *coli* thioredoxin (37°C), we can use, in the absence of a published experimental value, the half-life corresponding to the unfolding rate constant at 37°C (k_0_ = 1.84·10^−4^ min^-1^; τ = 1/k_U_ = 5432 min = 3.8 days), at least as a working hypothesis for the protein *in vivo* lifetime. We assume that this lifetime value has been conserved during evolution and estimate the ancestral environment temperatures as the temperatures at which a value of k_U_ of 1.84·10^−4^ min^-1^ is achieved for the laboratory resurrected proteins. The environmental temperature values thus obtained (Fig 11A) appear in reasonable agreement with the values derived from the isotopic composition of cherts and the denaturation temperatures of the Elongation Factors (unlike what is observed with the denaturation temperatures, which are much higher: Figs 1 and 2). One could argue that the half-life value derived from an *in vitro* unfolding rate may be a poor estimate of the *in vivo* lifetime and that, in any case, it appears unlikely that the lifetime value is conserved over 4 billion years. The point here, however, is that the calculation is extremely robust against choice of the lifetime value (as anticipated by the simulations of Figs 5 and 7). Thus, qualitatively the same result is seen in Fig 11B, where we provide a calculation identical to that of Fig 11A, except that for each protein we include the host environmental temperature interval corresponding to the 1 day to 2 months range in lifetime (i.e., a factor of 60). Even when using the comparatively large environmental temperature intervals corresponding to the one hour to one year range in lifetime (i.e., a factor of about 10^4^), the same general message is obtained: qualitative agreement with temperatures derived from isotopic analysis and support for the thermophilic character of ancient life (Fig 11B). In fact, it is fascinating that the calculations shown in Fig 11 are consistent with a minimum estimate (about 75°C) of the environmental temperature of the last universal common ancestor (LUCA) recently inferred from the properties of laboratory resurrections of nucleoside diphosphate kinases [1].

Certainly, the calculations shown in Fig 11 are based on the unfolding rates of oxidized thioredoxin. However, consideration of the effect of active-site disulfide reduction on rates does not change the main conclusions of the analysis (see “Reduced thioredoxin *versus* oxidized thioredoxin” of S1 File).

## Discussion

The scientific literature would benefit from greater discussions on the relationship between protein denaturation temperatures (T_m_) and the environmental temperatures of host organisms (T_ENV_). A general correlation between T_m_ and T_ENV_, especially among different sets of homologous proteins, is not expected to a first approximation [36], and is quickly ruled out by the observation that human proteins display a wide range of denaturation temperatures despite existing at a constant environmental temperature of 37°C. On the other hand, good T_m_ vs. T_ENV_ correlations have been reported for particular protein systems in different organisms [1, 10, 27] yet these display varying degrees of disparity between the T_m_ and T_ENV_ values. Denaturation temperatures for Elongation Factors reasonably match the growth temperatures of their host organisms [10], a result which, as discussed in the Introduction, may be consistent with the conformational flexibility expected for a protein whose biological function involves a variety of conformational changes. Other experimental T_m_ vs. T_ENV_ correlations are characterized by T_m_ values many degrees above the corresponding environmental temperatures of the host [1, 27, 35] an intriguing observation that poses a number of immediate (and, so far, unresolved) evolutionary questions.

Protein denaturation temperatures many degrees above host environmental temperatures seem to indicate that natural selection is not operating on denaturation temperatures. However, the existence of a T_m_ vs. T_ENV_ correlation suggests that, whichever stability-related property is subject to natural selection, its evolutionary changes are somehow mirrored by denaturation temperature values. The analyses reported here are consistent with the view that natural selection at least operates on the basis of protein kinetic stability, i.e., on the basis of stability-linked features that determine protein half-life *in vivo* and that can be assessed, at least to some extent, from *in vitro* denaturation rates. We have shown that threshold selection for kinetic stability rationalizes the intriguing experimental T_m_ vs. T_ENV_ correlations. Specifically, a good correlation between protein denaturation temperatures and environmental temperatures with T_m_ values many degrees above the T_ENV_ values is expected to occur when:

1. *in vitro* thermal denaturation is an equilibrium unfolding process;
2. protein half-life is determined by the activation free energy barrier for protein denaturation;
3. the kinetically-relevant transition state (the “structure at the top of the barrier”) is substantially unstructured and, consequently, mutation effects on barrier height are reflected in the equilibrium unfolding free energy and the denaturation temperature;
4. the threshold value of *in vivo* protein half-life associated with purifying selection remains reasonably constant throughout the set of homologs in the correlation (our analyses suggest that reasonable conservation of the half-life threshold, i.e., on the order of a magnitude or so, suffices to produce a T_m_ vs. T_ENV_ correlation).

Our analyses of this model suggest the following approximate relationship between protein denaturation temperature and environmental temperature:

$$T_{m}\approx T_{ENV}+\Delta T_{m}\left(\tau_{DEG}\right)$$ (14)

where the T_m_ shift with respect to the environmental temperature is, to a first approximation, a function of the *in vivo* lifetime (degradation half-life). Admittedly, different proteins in the same host show widely different *in vivo* lifetimes [51–54] and consequently have different shifts (different ΔT_m_ values) that ultimately generate different T_m_ values for the same environmental temperature. However, protein *in vivo* lifetimes likely reflect functional requirements with, for instance, enzymes involved in regulation showing short *in vivo* half-lives [55] and proteins involved in exceptionally stable essential cellular structures displaying long *in vivo* half-lives [54]. It is, therefore, plausible that *in vivo* lifetime is roughly conserved across a set of homologous proteins in different hosts (i.e., across a set of evolutionary-related proteins performing equivalent molecular tasks in different organisms), thus leading to an approximately constant T_m_ shift and to a T_m_↔T_ENV_ correlation for that given set of proteins.

**Fig 12:**
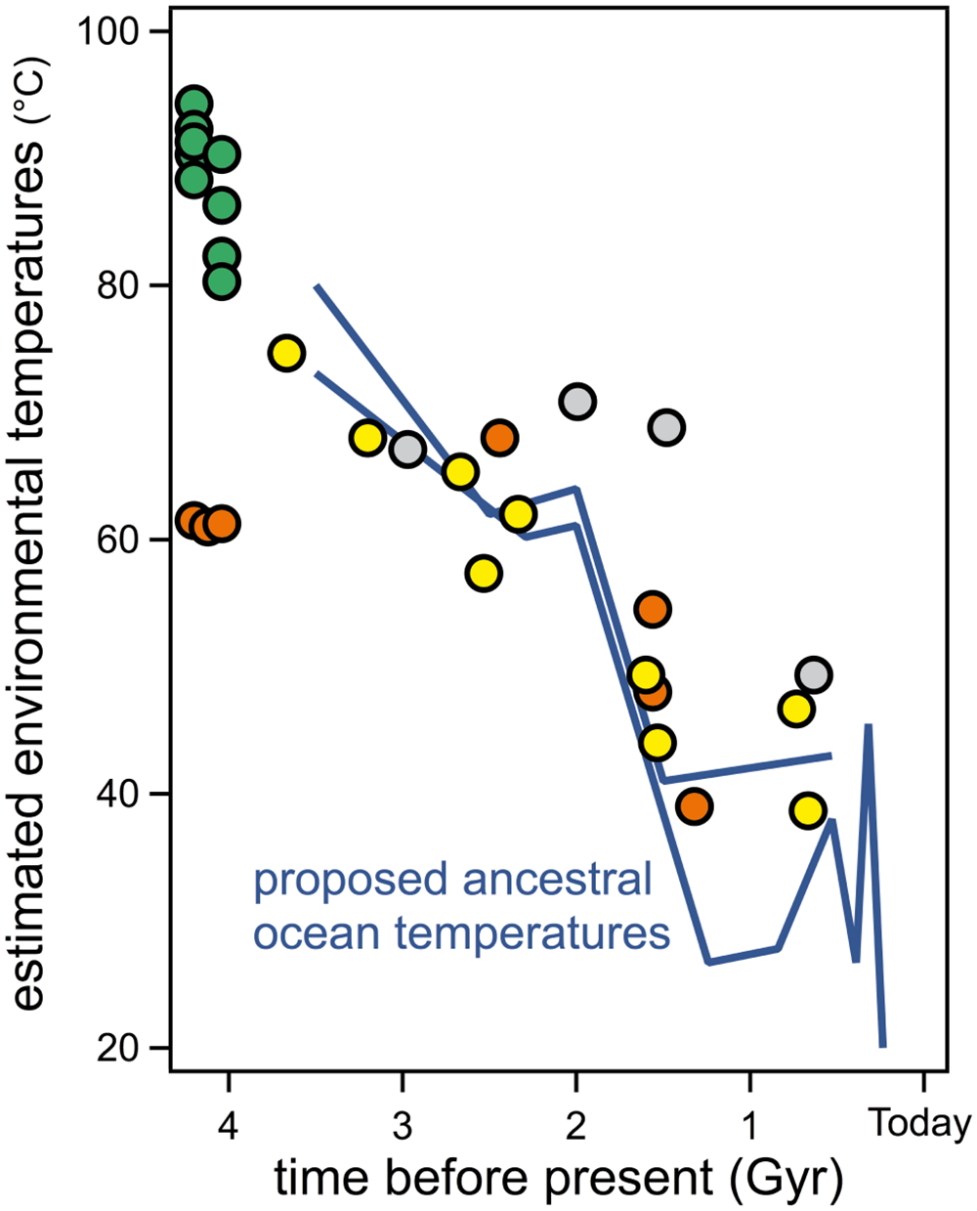
Simple estimation of ancestral environmental temperatures from the denaturation temperatures of resurrected Precambrian proteins. The color of the data symbols refers to the different protein families studied and is the same as that used in Fig 1.

Eq 14 predicts that the difference between T_m_-T_ENV_ should mainly reflect *in vivo* lifetime. A stringent test of this prediction would require knowledge of both the denaturation temperature and the *in vivo* lifetime for a substantial number of proteins. Unfortunately, our search in the literature has revealed little overlap between protein unfolding/denaturation databases (ProTherm: http://www.abren.net/protherm/) and published *in vivo* lifetime data [51–54]. Nevertheless, some degree of support for the model (points i-iv above and Eq 14) is derived from our analysis of the biophysical features of laboratory resurrections of Precambrian thioredoxins. In fact, we have shown these features to be consistent with the proposed model and, therefore, we have used the model to estimate T_ENV_ values for Archaean life from the temperature-dependent *in vitro* denaturation rates for the resurrected thioredoxins. The inferred environmental temperatures agree with the ancestral ocean temperatures derived from analysis of the isotopic composition of cherts (Fig 11) and are consistent with a recent estimate (above 75°C) of the environmental temperature for the last universal common ancestor [1]. Such an analysis of ‘ancestral denaturation temperatures’ can be taken one step further. Assuming the applicability of Eq 14 and that the *in vivo* lifetimes for lactamases, thioredoxins and nucleoside diphosphate kinases (NDK) are (roughly) conserved over long evolutionary time scales, we can use T_m_ shifts derived from extant protein data to derive (rough) estimates of ancestral environmental temperatures as T_ENV_ = T_m_-ΔT_m_. To this end, we used the denaturation temperatures of the extant *E*. *coli* proteins to calculate ΔT_m_ as T_m_ (*E*. *coli* protein)-37 (the results are ΔT_m_ = 19.4 degrees for Lactamases, ΔT_m_ = 52.0 degrees for Thioredoxins and ΔT_m_ = 18.7 degrees for NDK). As shown in Fig 12, the ancestral environmental temperatures thus estimated define a common trend for the four protein families, and these agree with the denaturation temperatures for Elongation Factors and the ancient ocean temperatures derived from the isotopic composition of cherts.

The consistency seen in Figs 11 and 12, however, should not be taken to imply that the specific scenario analyzed in this work (points i-iv above and Eq 14) describes the relationship between protein denaturation temperatures and organismal growth temperatures in *all* cases and, in fact, alternative scenarios are possible. Two of these scenarios are briefly described below.

We have assumed in our analysis that the kinetically-relevant transition state for protein denaturation is substantially unstructured (point iii in the scenario described above), in such a way that ϕ in Eq 11 is close to unity for many mutations and, consequently, that mutational effects on the activation barrier cause parallel changes in unfolding free energy and equilibrium denaturation temperature. There is little doubt that this description applies to thioredoxins (data reported in this work and [18]). Still, for other protein systems [68] the kinetically-relevant transition state may not be as "highly unstructured" as it is the case for thioredoxins. This does not necessarily imply that for these systems T_m_ does not correlates with T_ENV_. For instance, if, for most mutations, the value of ϕ has a value intermediate between 0 and 1, T_m_/T_ENV_ will likely be observed but with a modified slope. However, it is important to note that the unfolding/denaturation transition states for some proteins are substantially structured with a large activation free energy required for kinetic stabilization being related in these cases to the existence of a so-called “solvation barrier” [40, 41, 77, 78], meaning that, in the transition state, internal interactions (such as van der Waals interactions between buried hydrophobic residues) are partially broken or distorted but water molecules have not yet penetrated. These partially-unbroken and water-unsatisfied internal interactions contribute to the high activation free energy that confers kinetic stability onto the protein. However, with a transition state that, it is not only substantially structured but that also shows non-native features (a solvation barrier), the relation between mutational effects on unfolding kinetics and unfolding free energy may not be simple or straightforward. Accordingly, natural selection for kinetic stability (*in vivo* half-life) could lead to a correlation between environmental temperature and the *in vitro* rates of unfolding/denaturation, but the correlation between environmental temperatures and equilibrium denaturation temperatures may perhaps not hold in this case.

As suggested by experimental data on thioredoxin denaturation [16, 17, 59], we have assumed in our analysis that the *in vitro* denaturation temperature corresponds to an equilibrium two-state process (point i in the scenario described above). The presumption of two-state behavior is not critical for the outcome of the analysis because it can be easily shown [79] that the denaturation temperature value that describes a comparatively narrow, roughly symmetrical equilibrium unfolding transition is close to the temperature at which the constant for the native-unfolded equilibrium is at unity, even if there are significantly populated intermediate states. On the other hand, it must be recognized that the *in vitro* thermal denaturation of many proteins is a kinetically controlled process (see [41] and references therein) due to the occurrence of irreversible alterations of the unfolded or partially unfolded states (such as aggregation, for instance). In these cases, the value of the denaturation temperature calculated from a given experimental denaturation profile is not an equilibrium parameter but, rather, a measure of the temperature at which the half-life for irreversible denaturation matches the time-scale of the *in vitro* denaturation experiment. Irreversible alterations typically cause the denaturation temperature value to fall below the equilibrium value and may therefore increase the difference between environmental temperature and *in vitro* denaturation temperature. Furthermore, to the extent that the kinetically-relevant *in vivo* and *in vitro* transition states differ, *in vitro* irreversibility might contribute to blurring the correlation between environmental temperature and denaturation temperature for a set of homologs.

The alternative scenarios described above notwithstanding, our analysis and discussions reported here provide a general framework for understanding the evolutionary context for the relationship between the stability properties of proteins and the environmental temperatures of their host organisms. More specifically, this work suggests how future experimental work can address this relation in a meaningful way. It appears essential to explore the degree of evolutionary conservation of *in vivo* protein stability by determining the half-live values for sets homologous proteins from different organisms. Measurements of the in vitro denaturation rates for the same sets of proteins should help ascertain the extent to which in vivo stability correlates with protein kinetic stability as conventionally defined. Finally, determination of *in vitro* denaturation temperatures will immediately lead to a test of the proposed relationship between the *in vivo* half-lives and the discrepancy between denaturation temperature and host environmental temperature. These studies should help define the scope of applicability of the different scenarios discussed here and contribute to our overall understanding of the stability-related requirements for the multitude of proteins within any single organism.

## Materials and Methods

### Simulations of protein stability evolution that include threshold selection for thermodynamic stability and kinetic stability

We consider first an *in vitro* two-state equilibrium unfolding process, as shown in Eq 1. The temperature dependence of the unfolding equilibrium constant (defined in Eq 2) is given by the following integrated van´t Hoff equation:

$$lnK\left(T\right)=lnK\left(T_{0}\right)-\frac{\Delta H\left(T_{0}\right)-\Delta C_{P}T_{0}}{R}\left\{\frac{1}{T}-\frac{1}{T_{0}}\right\}+\frac{\Delta C_{P}}{R}ln\left(\frac{T}{T_{0}}\right)$$ (15)

where T_0_ is a reference temperature, taken to be 37°C (although this is unrelated to the fact that environmental temperature in many of our simulations is also 37°C). The initial values we used for the parameters on the left-side of Eq 1 are: lnK(T_0_) = -15.12, ΔH(T_0_) = 128.11 kJ/mol and ΔC_P_ = 5.8 kJ/mol (actually, the value of lnK(T_0_) is changed during simulations according to the protocol described further below). The unfolding heat capacity value is an experimental value for *E*. *coli* thioredoxin [16, 17] and straightforward calculations show that Eq 15 together with the initial values chosen for lnK(T_0_), ΔH(T_0_) and ΔC_P_ reproduce the values of the denaturation temperature (temperature at which K = 1) and the unfolding enthalpy at the denaturation temperature derived from experimental scanning calorimetry studies on *E*. *coli* thioredoxin unfolding: T_m_ = 89°C and ΔH(T_m_) = 430 kJ/mol [16, 17].

We next consider the protein in an *in vivo* environment in which irreversible alterations of nonnative states may take place. The two-state equilibrium shown in Eq 1 is then modified to include an irreversible denaturation step leading to a final state, F (proteolyzed protein, aggregated protein, etc.), as indicated in the Lumry-Eyring mechanism of Eq 4. Here, we carry out a full kinetic analysis of this mechanism to include the rates of folding (U→N), unfolding (N→U) and irreversible alteration (U→F). We assume the three processes are described by first-order equations with rate constants k_F_, k_U_ and k, respectively. The rate of irreversible denaturation (i.e., the rate of degradation) is then given by:

$$\frac{d\left[F\right]}{dt}=k\left[U\right]$$ (16)

We then make the reasonable assumption that the environmental temperature is below the protein denaturation temperature (T_ENV_<<T_m_) and, therefore, the concentration of the unfolded state is very small ([U]<<[N]+[F]). Consequently, we can apply the widely-used steady-state approximation of chemical kinetics to the unfolded state (if [U] is very low, then d[U]/dt is also very low and can be approximated as being equal to zero):

$$\frac{d\left[U\right]}{dt}=k_{U}\left[N\right]-k_{F}\left[U\right]-k\left[U\right]=0$$ (17)

from which the very low concentration of U present is given by:

$$\left[U\right]=\frac{k_{U}}{k_{F}+k}\left[N\right]$$ (18)

which can be substituted into Eq 16 to yield:

$$\frac{d\left[F\right]}{dt}=\frac{kk_{U}}{k_{F}+k}\left[N\right]$$ (19)

A rate constant for protein degradation can be defined through:

$$\frac{d\left[F\right]}{dt}=k_{DEG}\left[N\right]$$ (20)

and, using Eq 19,

$$k_{DEG}=\frac{kk_{U}}{k_{F}+k}$$ (21)

from which an expression for the degradation half-life time can be easily arrived at:

$$\tau =\frac{1}{k_{DEG}}=\frac{k_{F}+k}{kk_{U}}=\frac{1}{k_{U}}+\frac{1}{k\left(k_{U}/k_{F}\right)}=\frac{1}{k_{U}}\left\{1+\frac{k_{F}}{k}\right\}$$ (22)

Eq 6 is easily obtained from Eq 22 by taking into account that the ratio of the unfolding to folding rate constant equals the equilibrium constant (K = k_U_/k_F_). In fact, we assume in all our simulations that the rate of the irreversible step is very fast, so that k is much larger than k_F_, so that k_F_/k can be neglected in Eq 22 (or 1/kK can be neglected in Eq 6) and the degradation half-life is determined by the unfolding rate constant, as shown in Eq 8. See “Discussions on some approximations used in our simulations of protein stability evolution” of S1 File for further analysis.

According to Eq 8, the degradation half-life as a function of temperature can be calculated from the integrated Eyring equation that gives the temperature dependence of the unfolding rate constant:

$$ln\tau =-lnk_{U}\left(T\right)=-lnk_{U}\left(T_{0}\right)+\frac{\Delta H^{‡}\left(T_{0}\right)}{R}\left\{\frac{1}{T}-\frac{1}{T_{0}}\right\}-\frac{\Delta C_{P}^{‡}}{R}\left\{ln\left(\frac{T}{T_{0}}\right)-\left(\frac{T-T_{0}}{T}\right)\right\}$$ (23)

where ΔC_P_^‡^ is the activation heat capacity change, T_0_ is the reference temperature (37°C) and k_U_(T_0_) and ΔH^‡^(T_0_) are respectively the values of the unfolding rate constant and the activation enthalpy at the reference temperature. The initial values for the parameters in Eq 23 used in our simulations are those determined experimentally in this work for *E*. *coli* thioredoxin: lnk_U_(T_0_) = -8.26 (with k_U_ in min^-1^), ΔH^‡^(T_0_) = 168 kJ/mol and ΔC_P_^‡^ = 5.8 kJ·K^-1^·mol^-1^.

Each step in our simulation protocol involves introducing a mutation that changes the value of the unfolding free energy (ΔG) at the reference temperature. Mutational changes in ΔG (i.e. ΔΔG values) are randomly selected according to a very simple distribution that takes into account the well-known fact that most mutations in a protein are destabilizing (ΔΔG<0). Specifically, we assume the ΔΔG values to be uniformly distributed in a range between 2 and -5 kJ/mol, so that only about 30% of the mutations are stabilizing. This distribution is loosely suggested by the results of our previous study on the effect of 27 conservative mutations on the stability of *E*. *coli* thioredoxin [16, 17]. We considered the possibility of describing the ΔΔG distribution in terms of Gaussian functions (such as the two-Gaussian description used in [21]). However, in a long simulation involving a large number of steps, Gaussian ΔΔG distributions occasionally introduce mutations with unrealistically large effects, since the tails of a Gaussian function give non-zero probability to very high values of ΔΔG (or to negative ΔΔG with a very large absolute values); the simulation will then spend a considerable number of steps recovering from such unrealistic stability. It is important to note, in any case, that the exact form of the distribution used is not relevant to the outcome of the simulation, as long as it assumes the qualitatively correct proportion of stabilizing versus destabilizing mutations. Computational analyses based on protein structures and statistical analyses of experimental data [21, 22] indicate that the distribution of stability effects of mutations is approximately universal with most mutations (very roughly, about two thirds) being destabilizing [15–25]. The higher proportion of destabilizing mutations is, of course, consistent with the known scarcity of high stability sequences in proteins in general and with the known fact that proteins do not drift towards high stability but rather remain marginally above the evolutionary stability threshold. The distribution we have elected to use is simple and convenient from a computational point of view. Yet, it correctly takes into account that most mutations in a protein are destabilizing. Therefore, it correctly predicts that protein stability is marginal and that its change during evolution mostly reflects the corresponding changes in relevant stability thresholds for purifying selection. Furthermore, using a distribution based on experimental effects of conservative mutations makes sense, because such mutations are expected to occur more often during evolution and, indeed, conservative mutations tend to display the highest coefficients in substitution matrices, such as PAM [80] and BLOSUM [81]. Certainly, a distribution based on conservative mutations does not include highly destabilizing and highly stabilizing mutations. However, the former mutations will be rejected anyway in most cases and the latter mutations are rare and, typically, back-to-the-consensus or back-to-the-ancestor mutations in which the less energetically favored amino acid is fixed because some functional advantage. Therefore, such stabilizing mutations may decrease fitness for functional reasons: for a recent example see [12]. Still, we emphasize again that the relevant feature of the distribution we use is that it qualitatively includes the correct ratio of stabilizing vs. destabilizing mutations.

Once a ΔΔG at the reference temperature has been selected, modified values of the unfolding equilibrium constant at the reference temperature and the environmental temperature of the simulation are calculated through the successive application of Eq 3 (with T = T_0_) and Eq 15 (with T = T_ENV_). Also, once a transition-state ϕ-value has been decided upon (actually ϕ = 1 has been used in all the simulations), the mutational change in unfolding free energy barrier can be calculated from Eq 11. Then, modified values of the unfolding rate constant at the reference temperature and the degradation half-life at the environmental temperature of the simulation are obtained through the successive application of Eq 7 [k_0_ is taken as a constant [82] and cancels out in the calculation] and Eq 23. Finally, the calculated values of K and τ at the T_ENV_ allow the fitness to be determined (Eqs 4, 9 and 11) and a decision must be made regarding the acceptance or rejection of the mutation according to the criteria indicated in the main text (i.e., mutations that increase fitness are always accepted, mutations that decrease fitness are always rejected and mutations that do not change fitness are randomly accepted with a probability of 0.01).

All simulations were performed with lab-specific programs written in QB64, a self-hosting BASIC compiler that is a C++ emitter (http://www.qb64.net).

Further analysis is discussed in detail in “Discussions on some approximations used in our simulations of protein stability evolution” of S1 File.

### Protein preparation and experimental characterization

Purification of the thioredoxin variants studied in this work was carried out as previously described in detail [18, 47]. Stock solutions of proteins in aqueous buffer (50 mM Hepes, pH 7) and stock solutions of 7M guanidinium chloride in HEPES buffer were prepared as previously described [47].

Differential scanning calorimetry experiments were performed using a VP-DSC microcalorimeter (Microcal. Malvern) following procedures well-established in our lab and described in detail in previous publications [11, 16, 17, 47].

The kinetics of guanidine-induced unfolding were determined by steady-state fluorescence measurements, as previously described [47]. For each thioredoxin variant, several tens of kinetic runs were performed at different temperatures and guanidine concentrations. Fits of single-exponential equations to the kinetic profiles were visually excellent (see S3 Fig for representative examples). The unfolding rate constants derived from these fits were extrapolated to zero denaturant concentration using linear extrapolation and constant-ΔG extrapolation, as illustrated in Fig 7A and 7B for *E*. *coli* thioredoxin and LBCA thioredoxin. The extrapolations for the other thioredoxin variants studied in this work are including in the Supporting Information (S4–S11 Figs).

### Denaturation temperatures for the modern thioredoxins included in Fig 2 and growth temperatures for their host organisms

The denaturation temperatures of thioredoxins from *E*. *coli*, human cytosol and *Sulfolobus tokodaii* were taken from [11]. In order to include additional modern thioredoxins in Fig 2 we performed pubmed and google searches with the following queries: "thioredoxin stability & thermal unfolding", "thioredoxin thermal unfolding" and "thioredoxin melting temperature". We found experimental T_m_ data for thioredoxins from following organisms: *Bacillus acidocaldarius* [83], *Staphylococcus aureus* [84], *Chlamydomonas reinhardtii* [85] and pea [86] (note that T_m_ data for three isoforms of pea thioredoxin, *h1*, *h2* and *f*, are given by these authors, accounting for the fact that three pea data points are shown in Fig 2). In those cases in which several T_m_ values were reported for the same thioredoxin under different buffer conditions, the one corresponding to the pH value closest to 7 was used. Environmental temperatures for the modern thioredoxins included in Fig 2 were taken as the optimal growth temperatures of the host organisms (or the midpoint of the growth temperature range, when a range was provided) as reported in the following works: human [87], spinach [88, 89], pea [89, 90] and bacteria [91]. For both modern and ancestral proteins of Fig 2, r is 0.89 and p is 5.4·10_15_. Therefore, the correlation is statistically significant (a probability of 5·10_15_ of occurring by chance). The correlation is still significant if we include only the modern proteins (r = 0.93, p = 3·10_4_).

### Accessibility to solvent calculations

Unfolding changes in accessible surface (Table 1) were calculated as ΔASA = ASA(U)-ASA(N). Accessible surface areas for the native states were calculated from the known X-ray structures [92] while a tripeptide model was used to estimate the ASA values for the unfolded proteins.

Estimates of the accessibility to solvent in the transition state for thioredoxins denaturation (Table 1) were calculated as the rations of the activation values of m and ΔC_P_ to the corresponding equilibrium unfolding values. Unfolding equilibrium values used were m_EQ_ = 12.74 kJ·mol^-1^·M^-1^ [93] and ΔC_P,EQ_ = 5.8 kJ·mol^-1^ [94] which correspond to *E*. *coli* thioredoxin. Note, however, that, both denaturant m-values and unfolding heat capacities are known to scale with the corresponding changes in accessible surface areas [76, 95]. Since the calculated ΔASA values are essentially identical for all the thioredoxins studied here, we deem acceptable to use the *E*. *coli* thioredoxin values of m_EQ_ and ΔC_P,EQ_ for the calculation of transition-state accessibility in the ancestral thioredoxins.

## Supporting Information

S1 Fig Plot of logarithm of the equilibrium unfolding constant at the environmental temperature (37°C) versus simulation step for the simulations shown in the upper panel of Fig 4 in the main text. (PDF)

S2 Fig Plot of the final values for the unfolding equilibrium constant obtained in the simulations shown in the upper panel of Fig 7 of the main text *versus* the environmental temperature value used in the simulations. (PDF)

S3 Fig Representative examples of fluorescence intensity versus time profiles for the guanidine-induced unfolding of *E*. *coli* thioredoxin and several Precambrian thioredoxins at different temperaturas. (PDF)

S4 Fig Determination of the temperature-dependent unfolding rate constant for *E*. *coli* thioredoxin at zero guanidine concentration. (PDF)

S5 Fig Determination of the temperature-dependent unfolding rate constant for LAFCA thioredoxin at zero guanidine concentration. (PDF)

S6 Fig Determination of the temperature-dependent unfolding rate constant for LECA thioredoxin at zero guanidine concentration. (PDF)

S7 Fig Determination of the temperature-dependent unfolding rate constant for LGPCA thioredoxin at zero guanidine concentration. (PDF)

S8 Fig Determination of the temperature-dependent unfolding rate constant for LPBCA thioredoxin at zero guanidine concentration. (PDF)

S9 Fig Determination of the temperature-dependent unfolding rate constant for LACA thioredoxin at zero guanidine concentration. (PDF)

S10 Fig Determination of the temperature-dependent unfolding rate constant for AECA thioredoxin at zero guanidine concentration. (PDF)

S11 Fig Determination of the temperature-dependent unfolding rate constant for LBCA thioredoxin at zero guanidine concentration. (PDF)

S1 File Supplementary Analyses and Discussions. (PDF)
