# Supplementary material for: Selection for Protein Kinetic Stability Connects Denaturation Temperatures to Organismal Temperatures and Provides Clues to Archaean Life
Source: PLoS One. 2016 Jun 2;11(6):e0156657. doi: 10.1371/journal.pone.0156657 (PMC4890807; doi:10.1371/journal.pone.0156657)
Supplement: S1 Fig — (PDF) [file pone.0156657.s001.pdf]

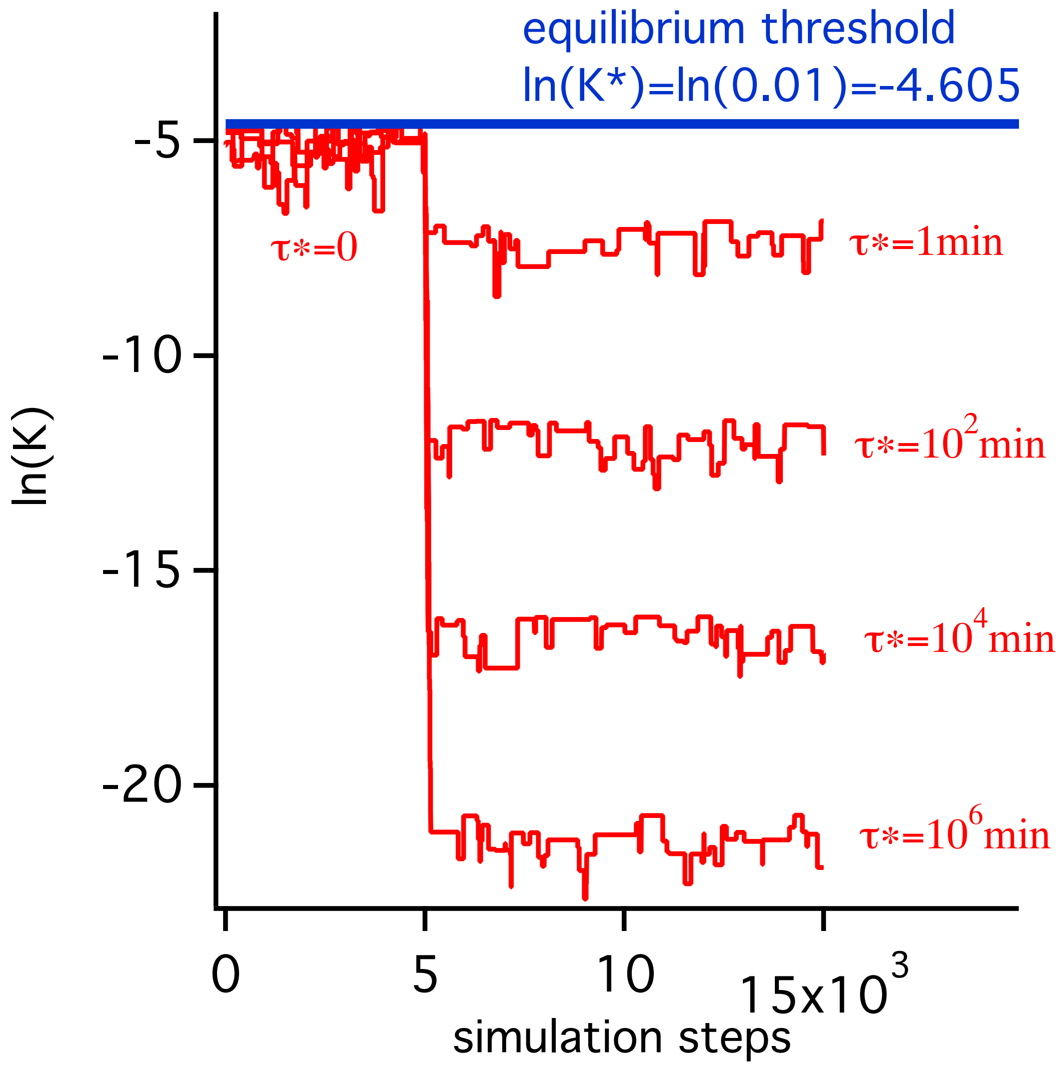

Fig. S1. Plot of logarithm of the equilibrium unfolding constant at the environmental temperature (37ºC) versus simulation step for the simulations shown in the upper panel of fig. 4 in the main text. The threshold values for the degradation half-life are shown. The plot illustrates the fact that, upon imposition of degradation threshold, the unfolding equilibrium constant is "dragged" to values well below the equilibrium threshold, which, as a result, does not determine the outcome of the simulations.
