# Supplementary material for: Selection for Protein Kinetic Stability Connects Denaturation Temperatures to Organismal Temperatures and Provides Clues to Archaean Life
Source: PLoS One. 2016 Jun 2;11(6):e0156657. doi: 10.1371/journal.pone.0156657 (PMC4890807; doi:10.1371/journal.pone.0156657)
Supplement: S2 Fig — (PDF) [file pone.0156657.s002.pdf]

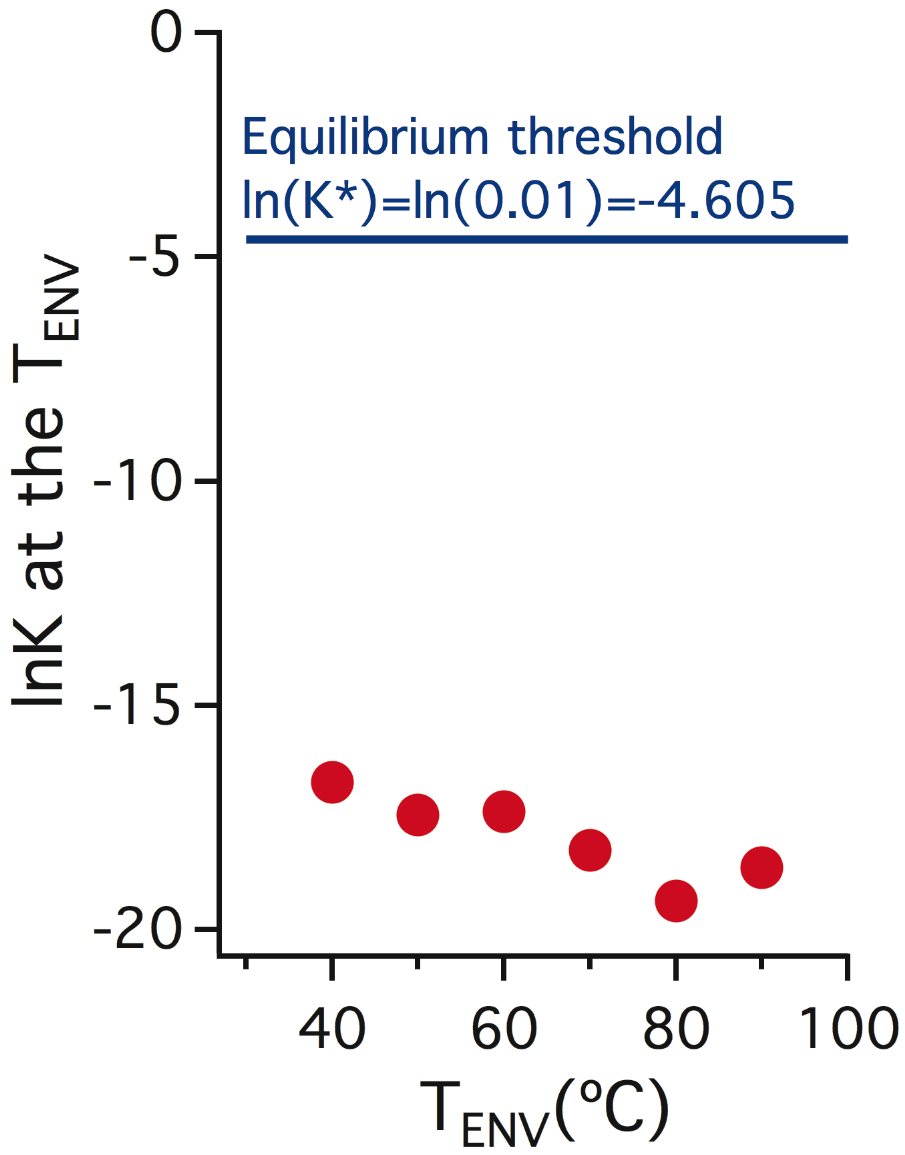

Fig. S2. Plot of the final values for the unfolding equilibrium constant obtained in the simulations shown in the upper panel of figure 7 of the main text *versus* the environmental temperature value used in the simulations. The plot illustrates the fact that, in these simulations, the unfolding equilibrium constant is "dragged" to values well below the equilibrium threshold, which, as a result, does not determine the outcome of the simulations.
