# Supplementary material for: Selection for Protein Kinetic Stability Connects Denaturation Temperatures to Organismal Temperatures and Provides Clues to Archaean Life
Source: PLoS One. 2016 Jun 2;11(6):e0156657. doi: 10.1371/journal.pone.0156657 (PMC4890807; doi:10.1371/journal.pone.0156657)
Supplement: S3 Fig — (PDF) [file pone.0156657.s003.pdf]

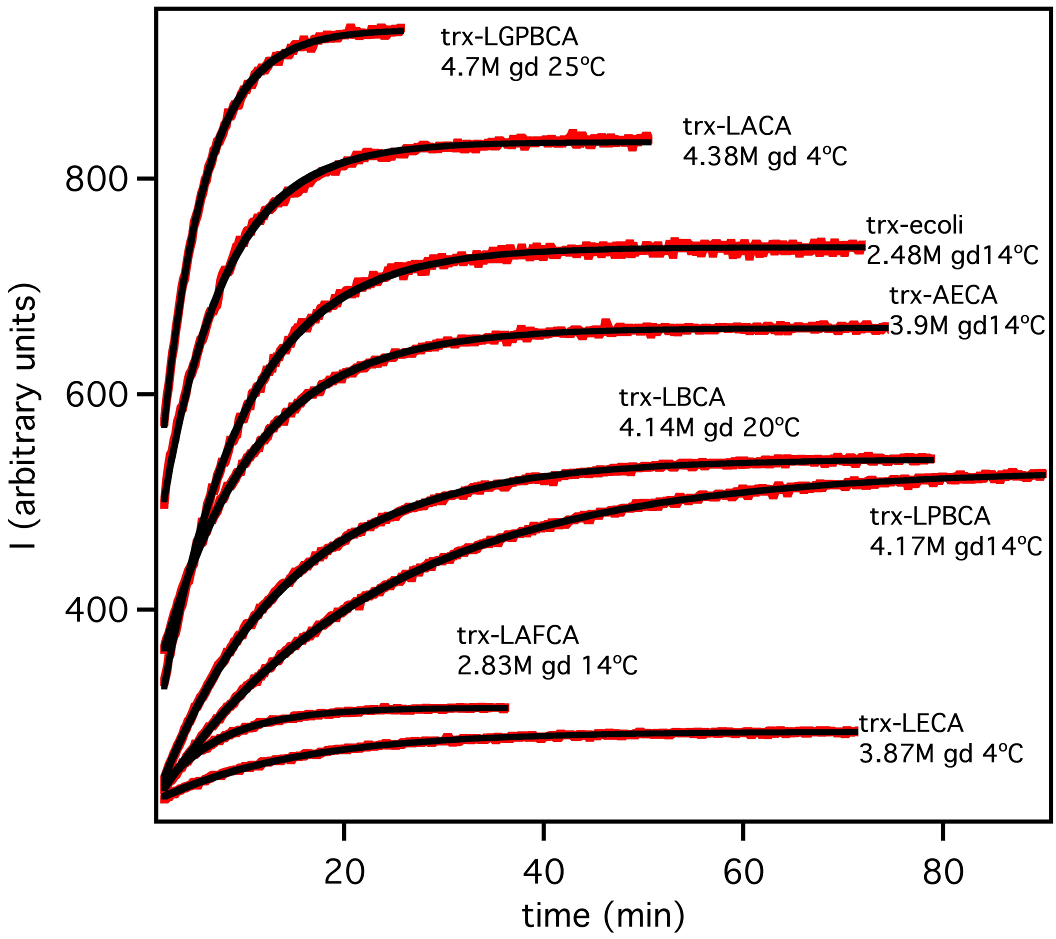

Fig. S3. Representative examples of fluorescence intensity versus time profiles for the guanidine-induced unfolding of *E. coli* thioredoxin and several Precambrian thioredoxins at different temperatures. Values of the unfolding rate constants (k_U) were derived from the fits of exponential functions to the corresponding fluorescence profiles. The numbers alongside the profiles stand for the temperature and the guanidine concentration used. Experimental data are shown in red and the black line represents the best fit of a single-exponential.
