# Supplementary material for: Selection for Protein Kinetic Stability Connects Denaturation Temperatures to Organismal Temperatures and Provides Clues to Archaean Life
Source: PLoS One. 2016 Jun 2;11(6):e0156657. doi: 10.1371/journal.pone.0156657 (PMC4890807; doi:10.1371/journal.pone.0156657)
Supplement: S6 Fig — (PDF) [file pone.0156657.s006.pdf]

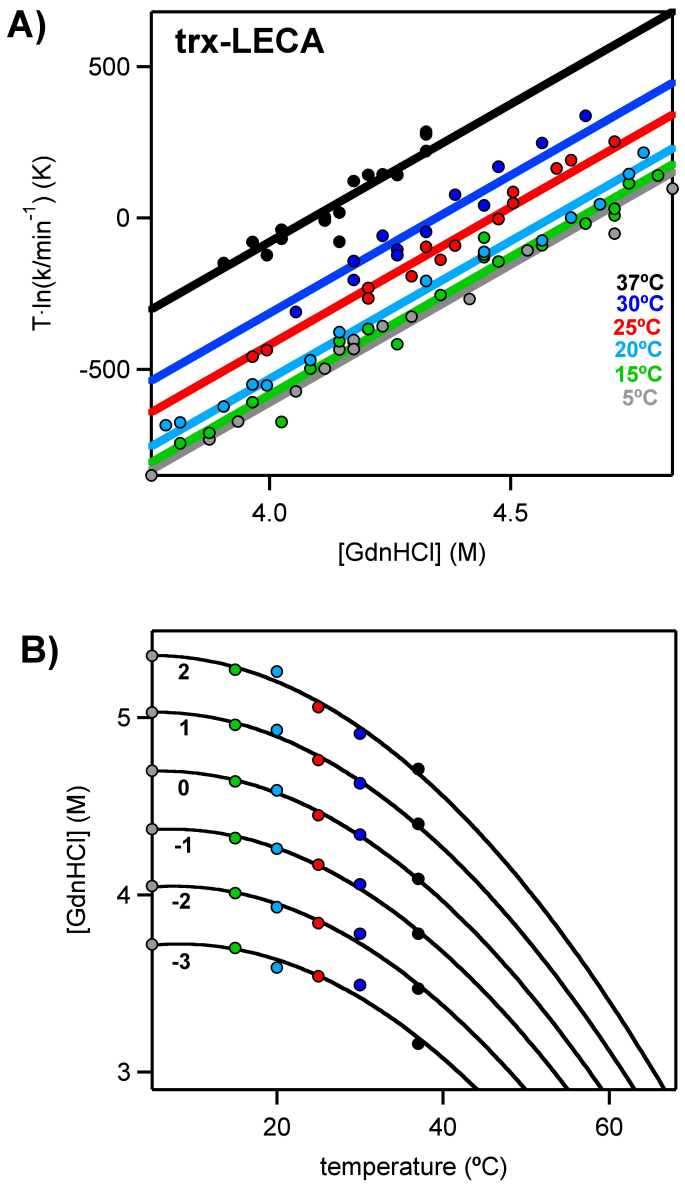

Fig. S6. Determination of the temperature-dependent unfolding rate constant for LECA thioredoxin at zero guanidine concentration. Guanidine-induced denaturation experiments were performed at several temperatures and guanidine concentrations. (A) Linear extrapolation to zero guanidine concentration to obtain the values of rate constants at lower temperatures. (B) Constant-lnk_U extrapolation to obtain the values of the rate constants at the higher temperatures. The color of the data points refers to the temperature, as shown in panel A. The lines are the best fits of second-order polynomials. The numbers alongside the lines stand for the lnk_U values.
