# Supplementary material for: Selection for Protein Kinetic Stability Connects Denaturation Temperatures to Organismal Temperatures and Provides Clues to Archaean Life
Source: PLoS One. 2016 Jun 2;11(6):e0156657. doi: 10.1371/journal.pone.0156657 (PMC4890807; doi:10.1371/journal.pone.0156657)
Supplement: S1 File — (PDF) [file pone.0156657.s012.pdf]

Supplementary Analyses and Discussions

(Supplementary data for *"Selection for Protein Kinetic Stability Connects Denaturation Temperatures to Organismal Temperatures and Provides Clues to Archaean Life"* by M. Luisa Romero-Romero, Valeria A. Risso, Sergio Martinez-Rodriguez, Eric A. Gaucher, Beatriz Ibarra-Molero, and Jose M. Sanchez-Ruiz)

CONTENT

Simple analysis of flux under thermodynamic and kinetic control of enzyme stability..........2

Discussion and some general comments on the proposed model of protein stability evolution..........6

Analysis of all plausible scenarios that could "save" the interpretation based on natural selection for thermodynamic stability..........9

Reduced thioredoxin *versus* oxidized thioredoxin..........15

Discussions on some approximations used in our simulations of protein stability evolution..........19

Simple Analysis of Flux under Thermodynamic and Kinetic Control of Enzyme Stability

We present here a very simple analysis of flux that should help clarify several aspects of the model explored in the main text and, in particular, the intuitive meaning of the thermodynamic and kinetic stability thresholds.

The flux through a metabolic pathway (F) is generally assumed to follow a saturation dependence (i.e., Michaelis-Menten-like) with the activity of an enzyme in the pathway (1, 2). For the purposes of the present analysis, it is convenient to write such dependence as:

$$F = \frac{[N]/_{\alpha}}{1+[N]/_{\alpha}} F_{MAX} \qquad \text{(S1)}$$

where, F_{MAX} is the maximum attainable flux, [N] is the concentration of functional (i.e., active or "native") enzyme and α is a constant equal to the concentration of functional enzyme at which the flux is half the maximum value. Figure A below shows the saturation plot predicted by equation S1.

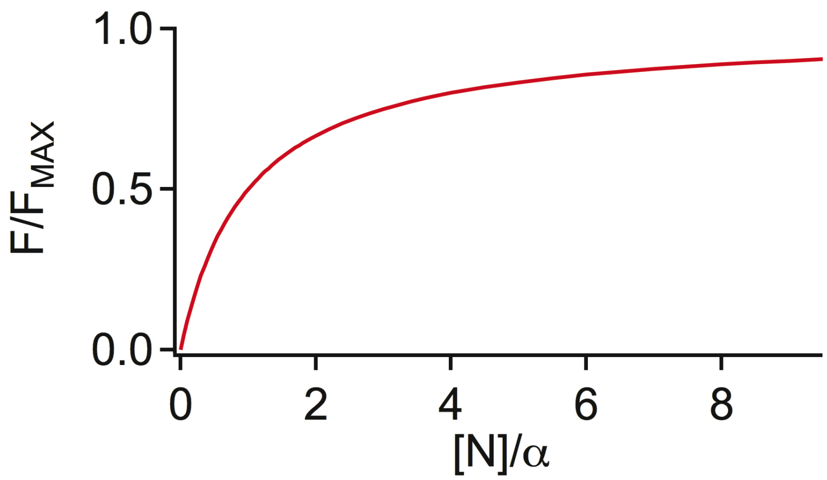

FIGURE A

Thermodynamic control of enzyme stability in the cell

We posit here that the stability of the folded protein in the cell is determined by thermodynamics. For two-state unfolding, this means that the concentration of native and unfolded protein are determined by the equilibrium unfolding constant:

$$K = \frac{[U]_{EQ}}{[N]_{EQ}} \qquad \text{(S2)}$$

$$[U]_{EQ} = \frac{K}{1+K}[E]_T \qquad \text{(S3)}$$

$$[N]_{EQ} = \frac{1}{1+K}[E]_T \qquad \text{(S4)}$$

where $[E]_T$ is the total enzyme concentration. We are not postulating here whether the enzyme is synthesized or degraded. We are assuming, however, that the rates of synthesis and degradation are slow compared with the time required for the unfolding N↔U equilibrium to be established. Accordingly, we take the total enzyme concentration, $[E]_T$, to be in steady state and the fractions of enzyme present as native (functional) and unfolded (non-functional) states are those given (equations S3 and S4) by the unfolding equilibrium constant (and, therefore, by the unfolding free energy change, since ΔG=-RTlnK).

Substitution of equation S4 into equation S1 (assuming $[N]=[N]_{EQ}$) followed by straightforward rearrangement leads to:

$$\frac{F}{F_{MAX}} = \frac{[E]_T/\alpha}{1+K+[E]_T/\alpha} \qquad \text{(S5)}$$

Figure B below shows plots of flux versus the value of the unfolding equilibrium constant for different values of the $[E]_T/\alpha$ ratio (numbers alongside the profiles).

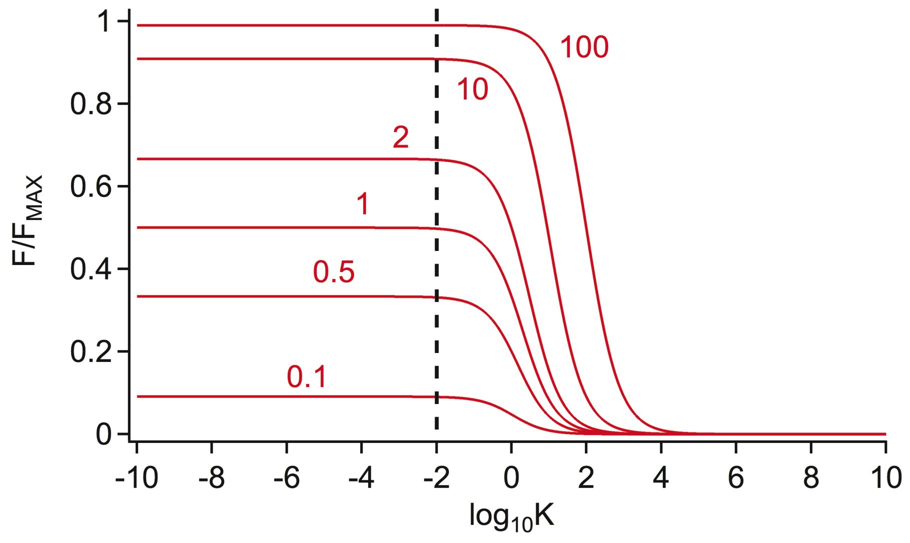

FIGURE B

The profiles in figure B are qualitatively consistent with "threshold behavior". That is, under the assumption that flux affects organismal fitness, they indicate that fitness will be compromised when the value of K becomes smaller than a certain value.

The profiles in figure B span three orders of magnitude of the $[E]_T/\alpha$ ratio (although we could expect on general grounds the total enzyme concentration *in vivo* to not be too different from the enzyme concentration, i.e., $[E]_T/\alpha \approx 1$) and the threshold appears to depend on the ratio value. We see from figure B that, for K values below roughly 10^{-2}, flux is independent of the value of K, regardless of the value of $[E]_T/\alpha$. This is reasonable since for $K=10^{-2}$ the fraction of enzyme which is in the native state is about 99% and further increases in K cannot significantly increase the amount of functional enzyme. Therefore, 10^{-2} is also a reasonable lower limit for the evolutionary threshold associated to the unfolding equilibrium constant (K*) under the assumption of thermodynamic control of the fraction of native protein. That is, values of K* higher than 10^{-2} (even up to unity) could be consistent with the "equilibrium scenario", but additional factors beyond natural selection for thermodynamic stability would be required to explain K* values significantly lower than 10^{-2} (in particular, since most mutations in a protein are destabilizing while drift towards high stability is unlikely to occur).

Strong kinetic control of enzyme stability in the cell

We posit here that the stability of the protein in the cell is determined by kinetics. That is, we do not take the unfolding equilibrium to be established. Rather, we assume that non-native (unfolded or partially unfolded) molecules are quickly degraded and, therefore, that the rate the degradation is given by the rate of unfolding ($k_U[N]$). We further assume that the level of enzyme in the cell is maintained by balance between the rate of synthesis (r_S) and the rate of degradation ($k_U[N]$):

$$\frac{d[N]}{dt} = r_S - k_U[N] = 0 \qquad \text{(S6)}$$

which gives,

$$[N] = \frac{r_S}{k_U} = r_S \cdot \tau = \alpha \frac{\tau}{\tau_{1/2}} \qquad \text{(S7)}$$

where τ ($=1/k_U$) would be the degradation half-life in the context of this simple model and we have defined $\tau_{1/2}$ as the half-live value at which $[N]=\alpha$ and the flux is half the maximum value. Substituting equation S7 into equation S1 yields:

$$\frac{F}{F_{MAX}} = \frac{\tau/\tau_{1/2}}{1+\tau/\tau_{1/2}} \qquad \text{(S8)}$$

Figure C shows the plot of flux versus degradation half-life predicted by equation S8.

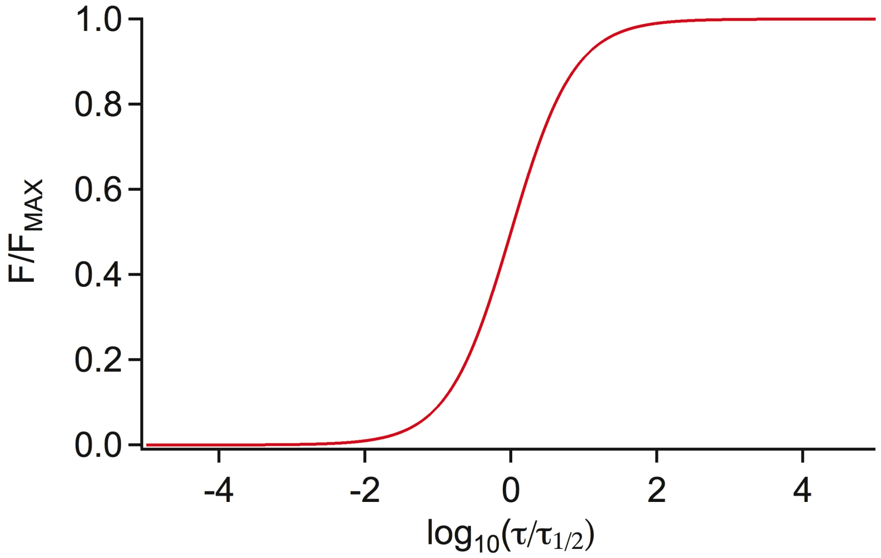

FIGURE C

The plot in figure C is also qualitatively consistent with threshold behavior. That is, for degradation half-lives above roughly $100 \cdot \tau_{1/2}$ flux is independent of the value of τ, while flux (and, consequently, organismal fitness) would decrease substantially for τ values below (roughly) $100 \cdot \tau_{1/2}$. Therefore, it seems reasonable, in this strong kinetic control scenario, to assume a threshold value for the degradation half-life (τ^* in the main text) although in this case the modeling does not provide any indication as to which values of τ^* are to be considered acceptable. In practice, we may expect τ^* values to span the experimental range of *in vivo* protein lifetimes, which is quite broad (see main text for details).

Discussion and some general comments on the proposed model of protein stability evolution

First of all, we notice that our proposed model for protein stability evolution is outside the context of population dynamics (although, it viewed through a "population dynamics lens" it would correspond to a monoclonal population in the limit of low mutation rate). Certainly, analyses based on population dynamics approaches have been very important in providing essential clues about the relation between organismal fitness and stability at the proteome level (3-5). Note, however, that the puzzling fact that we are trying to rationalize here is that the denaturation temperatures of a protein appear to remain about 50 degrees above the (changing) environmental temperatures over of geological time scale of billions of years. Actually, the time scale of Fig 1 of the main text is essentially the time span of life on Earth. Over this of time, not only populations changed to adapt to new environments, but also, species became extinct, new species appeared, eukaryotic cells arose, multicellularity evolved, etc. Note, furthermore, that the correlations between protein denaturation temperatures and environmental temperatures reported in the literature involve sets of homologs in different organisms (2, 6, 7) (see, also Fig 2 in the main text). That is, these correlations reflect again evolutionary time scales long enough to include speciation events. Overall, it is not clear to us how simple population dynamics analyses can be extended to the very long evolutionary time scales in a meaningful way. Our simple model is, therefore, outside the context of population dynamics, and, in fact, the fitness functions we use (equations 4 and 9 in the main text) are not, strictly speaking, meant to describe organismal fitness in the usual population dynamics sense. They are rather mathematical devices that allow us to impose threshold selection in the framework of our model.

Our proposed model is based upon the notion that the evolution of protein stability can be described to a substantial extent in terms of thresholds. That is, mutations that bring protein stability below a certain threshold are rejected. On the other hand, mutations that do not violate the threshold may be accepted, although that does not mean that a given protein will necessarily drift away from the stability threshold and become highly stable. In fact, a number of experimental and computational studies support that the distribution of stability effects in proteins follows an approximately universal distribution (3, 8) with most mutations being destabilizing (3, 8-17). Therefore, the

scarcity of high-stability sequences combined with the existence of a stability threshold leads to protein stability being typically marginal, i.e., slightly above the threshold. These ideas are well known and can be found, in one way or another, in many published papers on protein evolution. What is new in the approach reported here is the introduction of two stability thresholds, related to the fact that protein stability comes in two flavors: thermodynamic stability and kinetic stability. The existence of these two different thresholds can be intuitively justified on the basis of simple analyses of the interplay between protein production, degradation and stability in the cell, as described in "Simple models of protein stability evolution that include selection for thermodynamic stabilization and kinetic stabilization" of the main text and elaborated in some more detail above.

We admit, of course, that our approach is very simple and does not take into account a number of factors that may affect the relation between protein stability and organismal fitness. First of all, we have considered an equilibrium scenario and a strong kinetic control scenario in which the unfolding equilibrium constant and the unfolding rate, respectively, determine *in vivo* stability. However, intermediate situations in which the refolding rate plays an important role may also conceivably occur. Modeling refolding in this context, however, is not straightforward. Even for proteins whose equilibrium unfolding follows the two-state model, kinetics may not be two-state. A typical situation is that unfolding rates can be described in terms of single activated process, while refolding is kinetically complex involving several steps, significantly populated intermediate states or kinetic traps, etc. Secondly, and most important, the cytoplasm is known to be an active medium and, in particular, the protein quality control (PQC) system of the cell may modulate the effect of mutations on organismal fitness (13). Certainly, the role of the PQC can be included in computational modeling of protein homeostasis (18, 19) but, again, it is not at all clear whether (and how) these modeling efforts can extended to the very long evolutionary time scales in a meaningful way.

It should be clear from all the above that the model we introduced in the preceding section is not meant to provide an accurate depiction of the many factors that modulate the role of protein stability in organismal fitness in a population dynamics context. Rather, it is a simple framework that allows us to explore how natural selection for thermodynamic and kinetic stabilities shape the relation between protein denaturation temperatures and the environmental temperatures. The rationale behind our

approach is that, whichever factor is responsible for the conservation of the T_m-T_{ENV} difference over long evolutionary time scales (even over billions of years) should be robust enough to be revealed even by a very simple model. It is important to note, nevertheless, that we do not intend to derive evidence for our proposal (natural selection for kinetic stability) solely from the outcome of our dynamic simulations but, rather, from the coherence of the computational results with experimental data. This is the reason why this work includes a rather extensive experimental work on the temperature-dependent denaturation rates of a large number of resurrected Precambrian thioredoxins. It is the congruence between the experimental results on the kinetic stability of resurrected proteins and the results of the computational simulations that lends credence to our proposal, at least in the case of thioredoxins.

Analysis of all plausible scenarios that could "save" the interpretation based on natural selection for thermodynamic stability

The results shown in Fig 3 of the main text support that a difference of ~50 degrees between denaturation temperature and environmental temperature can hardly be explained in terms of natural selection for thermodynamic stability. However, it is important to discuss all plausible scenarios that could "save" the interpretation based on natural selection for thermodynamic stability. We do this in the following paragraphs:

<u>Scenario 1</u>: the unfolded state is highly detrimental for fitness. Between $K^*=0.01$ and $K^*=10^{-6}$ there is only a 1% difference in terms of the fraction of native protein, but there is a four orders of magnitude difference in terms of the fraction of unfolded protein. That unfolded states may be detrimental is suggested by the existence of the so-called "unfolded protein response" (20). If unfolded thioredoxin has a detrimental effect, it appears reasonable that its equilibrium concentration is kept very low. There is one problem with this interpretation, however. It is difficult to envision how the unfolded protein can be so detrimental that only about one unfolded molecule in equilibrium with a million native molecules (as required by $K^*=10^{-6}$) can be tolerated. Actually, it is unlikely that one million thioredoxin molecules are present at a given time in a single *E. coli* cell, as the concentration of thioredoxin in an *E. coli* cell has been estimated as 75 μM (21) and the cell volume has been reported to be within the range 0.44-1.79 μm^3 (22) yielding about 50000 thioredoxin molecules per cell, assuming a cell volume of 1 μm^3. It follows, therefore, that, with $K^*=10^{-6}$, most cells at a given time would not have a single unfolded thioredoxin molecule. It is difficult to imagine what could be so dangerous about unfolded thioredoxin that natural selection has ensured that there is not even a single unfolded molecule per cell at equilibrium.

<u>Scenario 2</u>: organisms (at least, organisms with non-regulated temperature) may live in a comparatively wide range of environmental temperatures. The idea here is that organisms could often experience temperatures that substantially differ from that for optimal growth and the stability of their proteins, therefore, must be "prepared" for such temperature variations. One might thus argue that the denaturation temperature for *E. coli* thioredoxin is about 89 ºC because occasionally (but not too rarely) *E. coli* faces an environmental temperature that is many degrees above 37 ºC and close to 89 ºC. The first problem with this proposal is that it would require all proteins in *E. coli* (not just thioredoxin) to have very high denaturation temperatures, which is not the case. The

second problem with this proposal is that thermal niches for bacteria (and viruses) are highly asymmetrical with fitness declining gradually upon temperature decrease but declining sharply upon temperature increase (4, 23-25). For *E. coli*, for instance, Lenski and coworkers (23) reported a thermal niche of 28.5-41 ºC (for an optimal growth temperature of about 37 ºC) and 41 ºC is still about 50 degrees below the denaturation temperature of *E. coli* thioredoxin. A similar case can be put forward with human thioredoxin. Its denaturation temperature is about 94 ºC and the temperature of the human body is regulated to be close to 37 ºC with increases of just a few degrees (fever) being linked to medical conditions.

Scenario 3: denaturation temperatures *in vitro* and *in vivo* may differ. Interactions with the crowded intracellular interior may affect protein stability and one possibility worth considering is that the cytoplasm is strongly destabilizing, at least for thioredoxin. In this scenario, the equilibrium denaturation temperature for, for instance, *E. coli* thioredoxin *in vivo* would be substantially lower than the *in vitro* value (about 89 ºC) and perhaps approach the environmental temperature (37 ºC for *E. coli*). The first problem with this proposal is that, to explain that the difference between the *in vitro* T_m and the environmental temperature remains at about 50 ºC over billions of years (Figs 1 and 2 of the main text). We would need to invoke that the destabilizing effect of the intracellular interior has remained approximately constant over billions of years, a hypothesis that appears unlikely and over-speculative. The most serious problem with the proposal is, however, that it is inconsistent with recent pioneering studies on in-cell protein stability (26-32). These studies have shown that intracellular environment can be stabilizing or destabilizing in a sequence-dependent manner but, also, that the effects are typically of a few degrees in terms of denaturation temperature (and of a few kJ/mol in terms of denaturation free energy). These changes may certainly be relevant to the understanding of protein function and interactions *in vivo*, but they are rather small compared with the ~50 ºC T_m vs. T_{ENV} difference we are trying to rationalize here.

Scenario 4: reduced thioredoxin is the catalytically competent form of this enzyme and its denaturation temperature may be lower than that of the oxidized form. Thioredoxin catalysis (33) involves the cycling of the enzyme between the oxidized and reduced states of the active site cysteines (C32 and C35 in the *E. coli* thioredoxin numbering). The reduced thioredoxin–$(SH)_2$ efficiently reduces disulfide bridges of other proteins, a process that involves its conversion into the oxidized thioredoxin-S_2 form. Reduced thioredoxin is then regenerated by the action of thioredoxin reductases.

Most biophysical data on thioredoxins (including the denaturation temperatures given in Figs 1 and 2 of the main text) have been obtained with the oxidized form (C32-C35 disulfide bridge), because it is more stable than the reduced form and, in fact, reduced thioredoxin-$(SH)_2$ is rapidly air oxidized to yield thioredoxin-$(S)_2$. However, reduced thioredoxin can be considered as the physiologically relevant form (34) and, it may be argued, it is its denaturation temperature that it must be compared with the environmental temperature. It would be conceivable, in fact, that, unlike the T_m value for thioredoxin-$(S)_2$ (Figs 1 and 2 in the main text), the T_m value for thioredoxin-$(SH)_2$ does approach the T_{ENV}. There are two points to make regarding this proposal. First, the fact that thioredoxin-$(SH)_2$ is the form that actually reduces protein disulfides does not mean that the *in vivo* concentration of the oxidized form is necessarily negligible (in particular, because the oxidized form is more stable). Many years ago, Holmgren and Fagerstedt (35) reported experimental evidence supporting extensive amounts of thioredoxin-S_2 in *E. coli* cells. More recently, (36) found thioredoxin oxidation in human keratinocytes linked to epidermal growth factor signaling and likely caused by the generated ROS (reactive oxygen species). Interestingly, Holmgren and coworkers (37) have found a similar pattern of thioredoxin oxidation caused by ROS in HeLa cells but linked in this case to the treatment with the anticancer drug SAHA (the histone deacetylase inhibitor suberoylanilide hydroxamic acid). In general, thioredoxin molecules may plausibly spend a significant fraction of their "*in vivo* lives" in the oxidized form and, accordingly, the stability of thioredoxin-S_2 may be one the factors that determine the total thioredoxin concentration in the cell. Therefore, it appears reasonable to assume that the stability of oxidized thioredoxin is relevant for organismal fitness even if reduced thioredoxin is the catalytically competent form. Secondly, but most important, although thioredoxin-$(SH)_2$ is substantially less stable than thioredoxin-S_2, the stability difference does not change the overall picture in the context of this work. About 20 years ago, Sturtevant and coworkers (38) reported that reduction of the active site disulfide decreased the denaturation temperature of *E. coli* thioredoxin by about 10 degrees and we have determined in this work (see "Reduced thioredoxin *versus* oxidized thioredoxin" below) that the ~10 degrees decrease in T_m upon disulfide reduction also holds for the resurrected ancestral thioredoxins. A simple visual inspection of Fig 3 in the main text shows that such a decrease in T_m would bring our estimated threshold equilibrium constant from about 10^{-6} to about 10^{-5}. However, all the

reasons we have adduced for the unlikelihood of natural selection for thermodynamic stability on the basis of $K^*=10^{-6}$ would still hold with $K^*=10^{-5}$.

Scenario 5: thioredoxin catalysis involves intermediate states of low denaturation temperature. We could, perhaps, conceive that the formation of some intermediate states during thioredoxin catalysis (mixed-disulfide intermediates, for instance) involves alterations that are highly destabilizing. Accordingly, we could propose that natural selection operates on the basis of the thermodynamic stability of those intermediates and that the high stability of thioredoxin is required to guarantee that the critical intermediates do not unfold. The problem with this proposal is that implicitly involves a link between stability and catalysis that is not supported by the available experimental data. Single-molecule studies have, in fact, demonstrated that thioredoxin catalysis and thioredoxin denaturation temperature can be modulated in a, to a large extent, independent manner (39). Furthermore, accumulation of disruptive hydrophobic-to-ionizable amino acid mutations in the thioredoxin molecule have been shown to decrease denaturation temperature by several tens of degrees without necessarily eliminating activity (40). These results obviously argue against thioredoxin catalysis involving low stability intermediates.

Scenario 6: thioredoxins are known to undergo association processes *in vitro,* which may distort the measured value of the denaturation temperature in a concentration-dependent manner. Certainly, T_m for *E. coli* thioredoxin has been reported to decrease with protein concentration, a dependency that has been interpreted in terms of dimerization of both, the native and the unfolded state (41). However, the effect is apparent at concentrations somewhat higher than those typically employed in DSC (see Table I in (41)) and it is on the order of a couple of degrees, clearly negligible when compared with the ~50 degrees T_m vs. T_{ENV} difference we are concerned here. Also, in our previous studies we checked that the denaturation temperatures for the resurrected ancestral thioredoxins in the bacterial branch were invariant within the approximate 0.1-2 mg/mL concentration range.

Scenario 7: what really determines protein stability *in vivo* is the folding rate. Proteins are synthesized *de novo* as unfolded polypeptide chains which are susceptible to aggregation and misfolding. It could be argued then that a sufficient fast folding rate is required to guarantee proper folding *in* vivo and selection for fast folding is somehow reflected in a very low value for the unfolding equilibrium constant. Leaving aside the obvious fact that this would not be a scenario of natural selection for thermodynamic

stability but, rather, of selection for fast folding kinetics (i.e., a kind of selection for kinetic stability), there are several problems with this proposal. First of all, unlike unfolding, folding often shows a complex kinetics involving intermediate states, but, even if we assume folding to be a simple kinetic process, it is not clear why effects on folding rate should be reflected in the unfolding equilibrium constant. In the simplest case, the rate of folding would be determined by the difference in free energy between the folding-unfolding transition state and the unfolded state. There is no reason for mutations that decrease the folding free energy barrier to also increase thermodynamic stability (the free energy difference between the unfolded and native states) unless, of course, we specifically consider mutations that "only" raise the free energy of the unfolded state and transition state is highly structured and native-like (which does not appear to be case with thioredoxin: see “The transition state that determines the kinetic stability of laboratory resurrections of Precambrian thioredoxins is substantially unstructured” in the main text). Second, *de novo* synthesis and protein stabilization *in vivo* are quite different phenomena with widely disparate time scales. Protein synthesis occurs in a time scale of seconds to minutes and efficient folding is assisted by a complex system of nascent chain-binding chaperones and downstream chaperones (42). These chaperone systems are evolutionary conserved in the three domains of life and likely represent one fundamental mechanism used by Nature to prevent aggregation and misfolding linked to protein synthesis. Another mechanism may be provided by the ribosome itself, which, according to recent single-molecule studies, may also act as a molecular chaperone and help promote efficient attainment of the native state (43). In any case, once the protein is correctly folded, *in vivo* stability requires that it remains in the functional state in a complex milieu that not only favors non-specific irreversible alterations, but in which specific protein degradation mechanisms exist. Furthermore, and most important, the protein must remain functional over a time scale (the *in vivo* life time of the protein) that it is typically much longer than the folding time. Clearly, the capability of the native state to survive *in vivo* over long time scales cannot be directly interpreted in terms of folding rates. It is interesting in this context that, as reported recently (44), the rate thioredoxin folding can be accelerated by four orders of magnitude through a single mutation that compromises function. The authors (44) concluded indeed that "our data provide evidence for the absence of a strong evolutionary pressure to achieve intrinsically fast folding rates, which is most likely a

consequence of proline isomerases and molecular chaperones that guarantee high *in vivo* folding rates and yields".

Overall, none of the scenarios discussed above seems able to save the interpretation based on natural selection for thermodynamic stability. Admittedly, a combination of several scenarios perhaps could, but this would involve a violation of Occam's razor, inasmuch as a very simple interpretation in terms on natural selection for kinetic stability is possible (see "Large discrepancies between protein denaturation temperatures and host environmental temperatures can be explained on the basis of natural selection for kinetic stability" in the main text).

Reduced thioredoxin *versus* oxidized thioredoxin

Most biophysical data on thioredoxins (including those reported in the main text of this work) are obtained with the more stable oxidized form. As we discuss in the main text in detail, however, thioredoxin catalysis involves the cycling of the enzyme between the oxidized and reduced states of the active site cysteines. It is important, therefore, to explore how our conclusions will be affected if the thermodynamic and kinetic stabilities of reduced thioredoxin are considered. To this end, we used differential scanning calorimetry to determine the denaturation temperatures of the reduced forms of several of our resurrected ancestral thioredoxins. Experiments were performed in 50 mM Hepes buffer pH 7 and in the presence of 1 mM dithiothreitol (DTT) to achieve the reduction of the active site disulfide bridge. Solutions were also deaerated and saturated with nitrogen gas to prevent oxidation. Although the presence of DTT caused distortions in the instrumental baseline, the transitions associated to the reduced thioredoxins could be easily detected and the corresponding denaturation temperatures determined. Disulfide bridge reduction causes the following changes in denaturation temperature (ΔT_m) values: -9.2 degrees (LPBCA thioredoxin), -10.0 degrees (LBCA thioredoxin), -13.0 degrees (AECA thioredoxin) and -10.5 degrees (LGPBCA thioredoxin). These decreases of about ten degrees agree with the effect reported in the literature for the reduction of the active disulfide on the stability of *E. coli* thioredoxin (ΔT_m=-11.0 degrees (38)). As we noted in the main text, a simple visual inspection of Fig 3 reveals that a decrease of about ten degrees in T_m would bring our estimated threshold for the unfolding equilibrium constant from about 10^{-6} to about 10^{-5} and all the reasons adduced for the unlikelihood of natural selection for thermodynamic stability on the basis of $K^*{\sim}10^{-6}$ would still hold with $K^*{\sim}10^{-5}$.

A different problem is to what extent the destabilizing effect of active-site disulfide reduction affects unfolding rates and whether this effect modifies any of our conclusions. To explore this issue we have first used Schellman equation (45) and the estimated unfolding enthalpies at the T_m values (calculated from the Kirchoff's equation and the energetic parameters given in the main text) to transform the determined ΔT_m values into estimates of the corresponding changes in unfolding free energy (i.e. unfolding $\Delta\Delta G$ values associated to disulfide reduction). The results are: -13.1 kJ/mol (*E. coli* thioredoxin), -9.2 kJ/mol (LPBCA thioredoxin), -10.0 kJ/mol (LBCA thioredoxin), -13.0 kJ/mol (AECA thioredoxin) and -15.2 kJ/mol (LGPBCA

thioredoxin). Secondly, we have considered two limiting scenarios: I) the active-site disulfide is in a region that remains structured (i.e., native-like) in the unfolding transition state; II) the active-site disulfide is in a region that is fully unfolded in the transition state for unfolding. Under scenario I, the destabilizing effect of disulfide reduction would affect the native state and the transition state, thus the unfolding rate constants would not change and nor would estimates of the environmental temperatures (Fig 11 in the main text). Under scenario II, on the other hand, disulfide reduction would differentially affect the native and transition states and the unfolding free energy barrier will decrease with the concomitant increase in unfolding rate. In this limiting case, transition state theory can be used to estimate the unfolding rates of the reduced ancestral thioredoxins from the unfolding rates for the oxidized thioredoxins we report in the main text (see Fig 11 in the main text):

$$lnk_U(reduced) = lnk_U(oxidized) - \frac{\Delta\Delta G}{RT} \qquad (S9)$$

The results are given in figure D below (it seems appropriate to assume that ΔΔG is temperature-independent for these illustrative calculations).

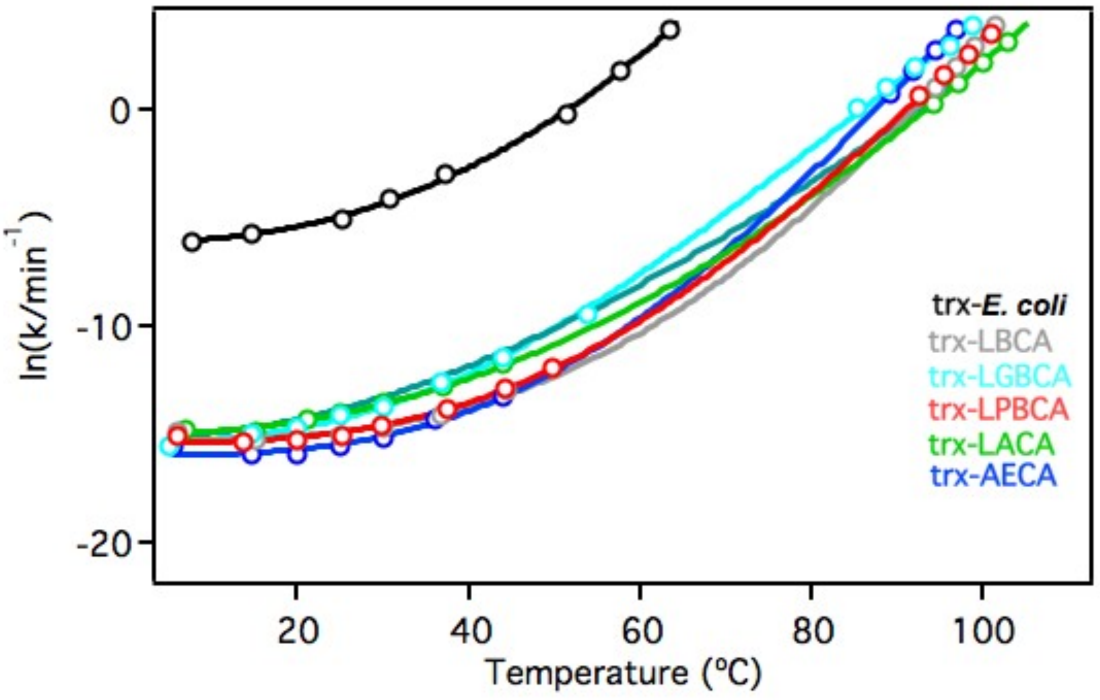

FIGURE D

Finally, we have repeated the calculations leading to the estimated environmental temperatures of Fig 11 in the main text but using this time the estimates of unfolding rates for ancestral thioredoxins given above. The results are given in figure E below:

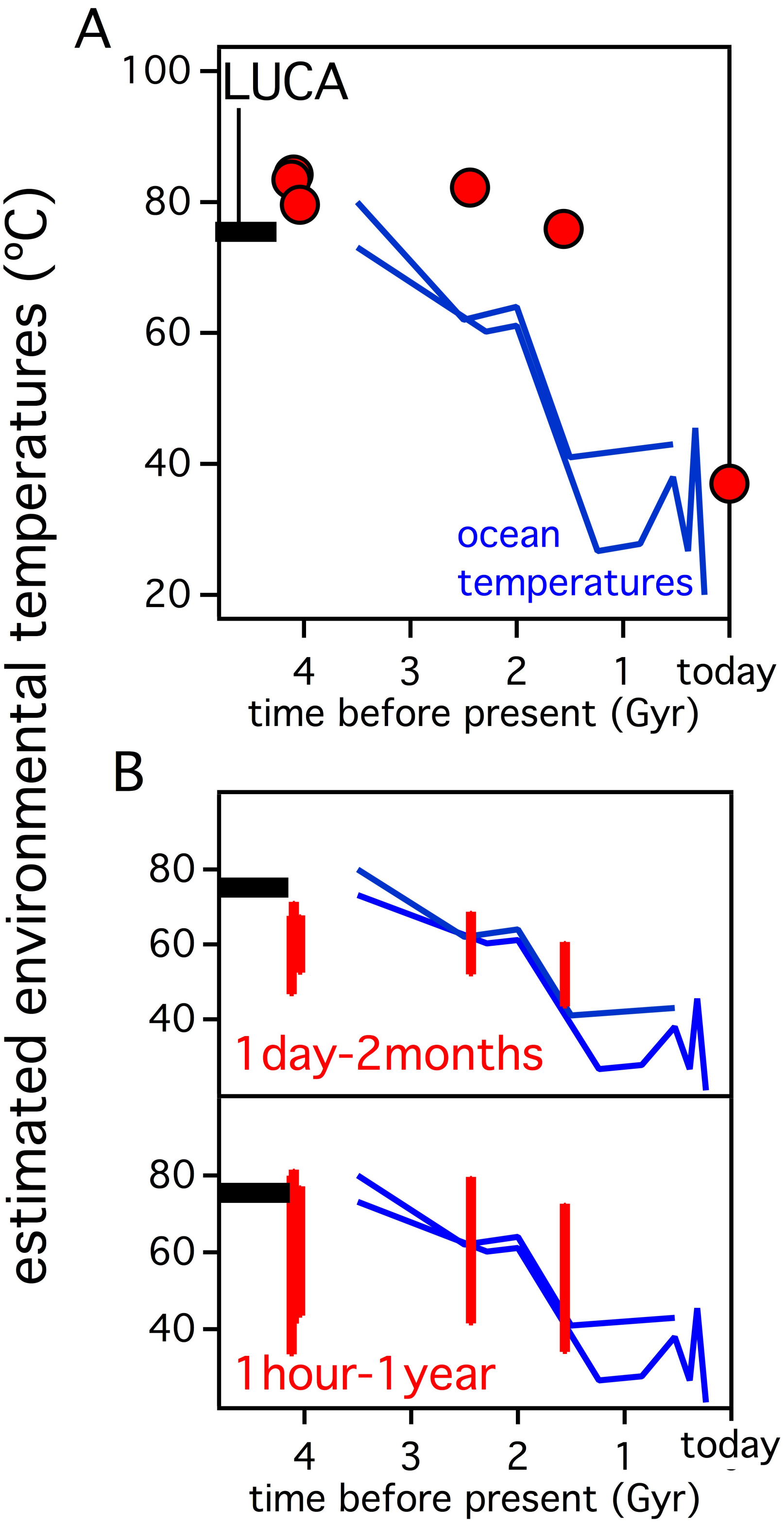

FIGURE E

Qualitatively, the interpretations of figure E above is the same as that conveyed by Fig 11 in the main text, although the T_{ENV} intervals appear larger, in particular for the ~4 billion year old proteins in the 1 hour-1 year range. In any case, it is important to note that the calculation summarized on figures D and E above correspond to the worst case scenario, in the sense that: I) we have assumed that all the destabilizing effect of disulfide reduction are reflected in the unfolding rate constant, while it cannot be ruled out that only a part of such destabilizing effects contribute to enhancing the unfolding rate constant; II) we have assumed that the rate of degradation is determined by the unfolding rate of the reduced protein, while it appears plausible that it is actually a weighted average of the rate of unfolding of the reduced and oxidized protein.

We overall conclude that specific consideration of the thermodynamic and kinetic stabilities of reduced thioredoxins do not substantially change the conclusions of this work.

Discussions on some approximations used in our simulations of protein stability evolution

Some of the approximations of the simulation procedure described in the main text require perhaps additional clarification. We do this in the following paragraphs:

- First, we assume in our simulations that the rate of the irreversible step is very fast, so that k is much larger than k_F, so that k_F/k can be neglected in equation 22 in the main text and the degradation half-life is determined by the unfolding rate constant, as shown in equation 8 in the main text. The alternative limiting case would be that k_{IRR} is much smaller than k_F and, therefore, that:

$$\tau = \frac{k_F}{k_U \cdot k} = \frac{1}{K \cdot k} \qquad \text{(S10)}$$

Both, equation 8 of the main text and equation S10 describe protein kinetic stability, the only difference between them is in the kinetically-relevant transition state (i.e., the highest point in the free energy profile). While in equation 8 the kinetically-relevant transition state lies between the native and the unfolded state, in equation S10 it lies between the unfolded state and the irreversibly denatured proteins. In principle, both situations could be plausible scenarios within the framework of natural selection for kinetic stability. We favor, however, the scenario embodied in equation 8 of the main text (unfolding is rate limiting) for a number of reasons. First, it is a well-known observation (46-48) that kinetic stability *in vitro* is often determined by the unfolding free energy barrier, a fact that is likely a reflection of the situation *in vivo*. Also, features that are conserved over billions of years (such as the ~50 degrees T_m vs. T_{ENV} for thioredoxins) are difficult to explain in terms of an equation (equation S10) that explicitly includes the overall rate of the processes responsible for irreversible denaturation because such processes likely change (in an unknown manner) over long evolutionary time scales. Finally, a high unfolding free energy barrier is a kind of "general safety device" that protects a protein against all types of irreversible processes that take place from the "unfolded side" of the barrier (from unfolded or partially unfolded states). It then appears reasonable that a high unfolding free energy barrier is the naturally selected strategy for generating kinetically stable proteins (47). Equation 8 in the main text is certainly consistent with this view.

- Secondly, we use energetic (or activation) parameters of wild-type *E. coli* thioredoxin in the equations that give the temperature dependence of the unfolding equilibrium constant (or the unfolding rate constant) and the reader may have perhaps

concluded that we are assuming that the unfolding enthalpies are not affected by mutation. This is not the case. We only assume that the mutational enthalpic effects are negligible when compared with the unfolding enthalpy value. That this should be a reasonable approximation in most cases is a consequence of well-known features of protein unfolding energetics. Consider the breakdown of unfolding free energy change in its enthalpic and entropic components: $\Delta G=\Delta H-T\Delta S$. Both ΔH and $T\Delta S$ are typically on the order of hundreds of kJ/mol (at temperatures not too close to the enthalpy inversion temperature) and cancel each other out to a substantial extent in the ΔG value, which is typically on the order of a few tens of kJ/mol (see, for instance (49)). Consider now the corresponding breakdown of the mutational effect on unfolding free energy, $\Delta\Delta G=\Delta\Delta H-T\Delta\Delta S$, where $\Delta\Delta G$ is typically on the order of a few kJ/mol and, therefore, comparable to the ΔG value. Assume now that all the mutational effects are of enthalpic origin: $T\Delta\Delta S=0$ and $\Delta\Delta H=\Delta\Delta G$. $\Delta\Delta H$ will then be on the order of a few kJ/mol and therefore, very small when compared with the typical ΔH values (on the order of hundreds of kJ/mol). Overall, whether mutation effects are mostly enthalpic ($\Delta\Delta H=\Delta\Delta G$) or mostly entropic ($\Delta\Delta H=0$) is largely inconsequential in terms of determining the temperature dependence of the unfolding equilibrium constant and the unfolding free energy. In fact, this is the basis of the well-known and widely-used method to calculate mutational $\Delta\Delta G$'s from the mutational effects on T_m proposed by John Schellman many years ago (45). We could envision mutations for which both $\Delta\Delta H$ and $T\Delta\Delta S$ are large and cancel each other out to a substantial extent to yield a small $\Delta\Delta G$ value. However, since unfolding enthalpies reflect structural features (accessible surface areas in the native structure; see, for instance (49)), most such mutations are expected to be disruptive, highly destabilizing and likely rejected during the course of evolution (and, therefore, whether their effect on the temperature dependencies is accurately described or not would be immaterial). Overall, neglecting mutational effects on enthalpies is reasonable and consistent with known features of protein energetics and, when combined with the assumption (supported by the data reported in this work and by our previous work on thioredoxins: (12, 50)) of a substantially unstructured transition state, predicts a correlation between denaturation temperature and unfolding rate constants at low temperature (panel D in Fig 7 in the main text), which is the result experimentally observed with the resurrected ancestral thioredoxins (Fig 10 in the main text). It is also worth noting in this context that our extensive DSC studies on LPBCA

thioredoxin and several single-mutant variants of this protein indicate that the unfolding energetics of LPBCA thioredoxin, in particular at the enthalpy and heat capacity levels, is congruent with that of *E. coli* thioredoxin, despite the large number of mutational differences between the proteins.

- As an additional clarification, one may have noticed that we use $\phi=1$ (equation 11 in the main text) in all our simulations and perhaps concluded that we are assuming the kinetically-relevant transition state is fully unfolded and that, therefore, our analysis implies that the rate of folding is strictly constant. However, a fully unfolded transition state would be identical with the unfolded state and imply that there is no barrier for folding. Although some small fast-folding proteins may be near (or at) the barrier-less, downhill folding regime, this is not the case for thioredoxin, as we noted previously (51). That the transition state for thioredoxin unfolding is substantially unstructured is supported by the metrics of solvent exposure determined in this work (see Table 1 in the main text) and by our previous mutational analyses (12). Yet, the transition state necessarily contains a small, highly-stable structured region that contributes to a high free energy over the native and unfolded protein. Such states with a small structured region have been described, for instance, in the irreversible denaturation of engineered phytases (52) and the misfolding of the prion protein (53). We expect most mutations in the thioredoxin molecule to occur in positions corresponding to the larger, unstructured regions in the transition state. Furthermore, we find plausible that the (comparatively rare) mutations that affect the critical structured region in the transition state are disruptive and are, therefore, rejected during evolution. Overall, we feel justified in assuming $\phi=1$ in our thioredoxin simulations. This, however, does not mean that we assume that there are no mutational effects on the folding rates. It is important to note that, while unfolding is often (but not always; see (54)) kinetically simple, folding is typically a complex process, even *in vitro*. This is clearly revealed by the fact that most chevron plots reported in the literature display roll-overs in the folding branch (indicating complexity in the folding kinetics, typically described in terms of populated intermediate states), while the unfolding branch is often lineal. Certainly, folding kinetics in *E. coli* thioredoxin has been experimentally shown to be highly complex and to involve the accumulation of kinetic intermediates (see, for instance (55)). In this scenario, even mutations in regions that are unstructured in the main transition state for unfolding could potentially affect the folding rate.

References

1. Kaltenbach M, Tokuriki N. Generation of effective libraries by neutral drift. Methods Mol Biol. 2014;1179:69-81.
2. Hart KM, Harms MJ, Schmidt BH, Elya C, Thornton JW, Marqusee S. Thermodynamic system drift in protein evolution. PLoS Biol. 2014;12(11):e1001994.
3. Zeldovich KB, Chen P, Shakhnovich EI. Protein stability imposes limits on organism complexity and speed of molecular evolution. Proc Natl Acad Sci U S A. 2007;104(41):16152-7.
4. Chen P, Shakhnovich EI. Thermal adaptation of viruses and bacteria. Biophys J. 2010;98(7):1109-18.
5. Wylie CS, Shakhnovich EI. A biophysical protein folding model accounts for most mutational fitness effects in viruses. Proc Natl Acad Sci U S A. 2011;108(24):9916-21.
6. Gaucher EA, Govindarajan S, Ganesh OK. Palaeotemperature trend for Precambrian life inferred from resurrected proteins. Nature. 2008;451(7179):704-7.
7. Akanuma S, Nakajima Y, Yokobori S, Kimura M, Nemoto N, Mase T, et al. Experimental evidence for the thermophilicity of ancestral life. Proc Natl Acad Sci U S A. 2013;110(27):11067-72.
8. Tokuriki N, Stricher F, Schymkowitz J, Serrano L, Tawfik DS. The stability effects of protein mutations appear to be universally distributed. J Mol Biol. 2007;369(5):1318-32.
9. Godoy-Ruiz R, Perez-Jimenez R, Ibarra-Molero B, Sanchez-Ruiz JM. Relation between protein stability, evolution and structure, as probed by carboxylic acid mutations. J Mol Biol. 2004;336(2):313-8.
10. Taverna DM, Goldstein RA. Why are proteins marginally stable? Proteins. 2002;46(1):105-9.
11. Godoy-Ruiz R, Perez-Jimenez R, Ibarra-Molero B, Sanchez-Ruiz JM. A stability pattern of protein hydrophobic mutations that reflects evolutionary structural optimization. Biophys J. 2005;89(5):3320-31.
12. Godoy-Ruiz R, Ariza F, Rodriguez-Larrea D, Perez-Jimenez R, Ibarra-Molero B, Sanchez-Ruiz JM. Natural selection for kinetic stability is a likely origin of

correlations between mutational effects on protein energetics and frequencies of amino acid occurrences in sequence alignments. J Mol Biol. 2006;362(5):966-78.
13. Bershtein S, Segal M, Bekerman R, Tokuriki N, Tawfik DS. Robustness-epistasis link shapes the fitness landscape of a randomly drifting protein. Nature. 2006;444(7121):929-32.
14. Kellogg EH, Leaver-Fay A, Baker D. Role of conformational sampling in computing mutation-induced changes in protein structure and stability. Proteins. 2011;79(3):830-8.
15. Tokuriki N, Tawfik DS. Stability effects of mutations and protein evolvability. Curr Opin Struct Biol. 2009;19(5):596-604.
16. Potapov V, Cohen M, Schreiber G. Assessing computational methods for predicting protein stability upon mutation: good on average but not in the details. Protein Eng Des Sel. 2009;22(9):553-60.
17. Sanchez IE, Tejero J, Gomez-Moreno C, Medina M, Serrano L. Point mutations in protein globular domains: contributions from function, stability and misfolding. J Mol Biol. 2006;363(2):422-32.
18. Powers ET, Powers DL, Gierasch LM. FoldEco: a model for proteostasis in E. coli. Cell Rep. 2012;1(3):265-76.
19. Bershtein S, Serohijos AW, Bhattacharyya S, Manhart M, Choi JM, Mu W, et al. Protein Homeostasis Imposes a Barrier on Functional Integration of Horizontally Transferred Genes in Bacteria. PLoS Genet. 2015;11(10):e1005612.
20. Walter P, Ron D. The unfolded protein response: from stress pathway to homeostatic regulation. Science. 2011;334(6059):1081-6.
21. Jacob C, Kriznik A, Boschi-Muller S, Branlant G. Thioredoxin 2 from Escherichia coli is not involved in vivo in the recycling process of methionine sulfoxide reductase activities. FEBS Lett. 2011;585(12):1905-9.
22. Kubitschek HE, Friske JA. Determination of bacterial cell volume with the Coulter Counter. J Bacteriol. 1986;168(3):1466-7.
23. Bronikowski AM, Bennett AF, Lenski RE. Evolutionary adaptation to temperature. VIII. Effects of temperature on growth rate in natural isolates of Escherichia coli and Salmonella enterica from different thermal environments. Evolution. 2001;55(1):33-40.

24. Cullum AJ, Bennett AF, Lenski RE. Evolutionary adaptation to temperature. IX. Preadaptation to novel stressful environments of Escherichia coli adapted to high temperature. Evolution. 2001;55(11):2194-202.
25. Knies JL, Izem R, Supler KL, Kingsolver JG, Burch CL. The genetic basis of thermal reaction norm evolution in lab and natural phage populations. PLoS Biol. 2006;4(7):e201.
26. Sarkar M, Smith AE, Pielak GJ. Impact of reconstituted cytosol on protein stability. Proc Natl Acad Sci U S A. 2013;110(48):19342-7.
27. Wirth AJ, Gruebele M. Quinary protein structure and the consequences of crowding in living cells: leaving the test-tube behind. Bioessays. 2013;35(11):984-93.
28. Monteith WB, Pielak GJ. Residue level quantification of protein stability in living cells. Proc Natl Acad Sci U S A. 2014;111(31):11335-40.
29. Guzman I, Gelman H, Tai J, Gruebele M. The extracellular protein VlsE is destabilized inside cells. J Mol Biol. 2014;426(1):11-20.
30. Guzman I, Gruebele M. Protein folding dynamics in the cell. J Phys Chem B. 2014;118(29):8459-70.
31. Konig I, Zarrine-Afsar A, Aznauryan M, Soranno A, Wunderlich B, Dingfelder F, et al. Single-molecule spectroscopy of protein conformational dynamics in live eukaryotic cells. Nat Methods. 2015;12(8):773-9.
32. Smith AE, Zhang Z, Pielak GJ, Li C. NMR studies of protein folding and binding in cells and cell-like environments. Curr Opin Struct Biol. 2015;30:7-16.
33. Holmgren A. Thioredoxin. Annu Rev Biochem. 1985;54:237-71.
34. Huber D, Chaffotte A, Eser M, Planson AG, Beckwith J. Amino acid residues important for folding of thioredoxin are revealed only by study of the physiologically relevant reduced form of the protein. Biochemistry. 2010;49(41):8922-8.
35. Holmgren A, Fagerstedt M. The in vivo distribution of oxidized and reduced thioredoxin in Escherichia coli. J Biol Chem. 1982;257(12):6926-30.
36. Halvey PJ, Watson WH, Hansen JM, Go YM, Samali A, Jones DP. Compartmental oxidation of thiol-disulphide redox couples during epidermal growth factor signalling. Biochem J. 2005;386(Pt 2):215-9.
37. Ungerstedt J, Du Y, Zhang H, Nair D, Holmgren A. In vivo redox state of human thioredoxin and redox shift by the histone deacetylase inhibitor

suberoylanilide hydroxamic acid (SAHA). Free Radic Biol Med. 2012;53(11):2002-7.
38. Ladbury JE, Kishore N, Hellinga HW, Wynn R, Sturtevant JM. Thermodynamic effects of reduction of the active-site disulfide of Escherichia coli thioredoxin explored by differential scanning calorimetry. Biochemistry. 1994;33(12):3688-92.
39. Rodriguez-Larrea D, Perez-Jimenez R, Sanchez-Romero I, Delgado-Delgado A, Fernandez JM, Sanchez-Ruiz JM. Role of conservative mutations in protein multi-property adaptation. Biochem J. 2010;429(2):243-9.
40. Garcia-Seisdedos H, Ibarra-Molero B, Sanchez-Ruiz JM. How many ionizable groups can sit on a protein hydrophobic core? Proteins. 2012;80(1):1-7.
41. Ladbury JE, Wynn R, Hellinga HW, Sturtevant JM. Stability of oxidized Escherichia coli thioredoxin and its dependence on protonation of the aspartic acid residue in the 26 position. Biochemistry. 1993;32(29):7526-30.
42. Hartl FU, Hayer-Hartl M. Molecular chaperones in the cytosol: from nascent chain to folded protein. Science. 2002;295(5561):1852-8.
43. Kaiser CM, Goldman DH, Chodera JD, Tinoco I, Jr., Bustamante C. The ribosome modulates nascent protein folding. Science. 2011;334(6063):1723-7.
44. Roderer DJ, Scharer MA, Rubini M, Glockshuber R. Acceleration of protein folding by four orders of magnitude through a single amino acid substitution. Sci Rep. 2015;5:11840.
45. Becktel WJ, Schellman JA. Protein stability curves. Biopolymers. 1987;26(11):1859-77.
46. Sanchez-Ruiz JM. Theoretical analysis of Lumry-Eyring models in differential scanning calorimetry. Biophys J. 1992;61(4):921-35.
47. Sanchez-Ruiz JM. Protein kinetic stability. Biophys Chem. 2010;148(1-3):1-15.
48. Plaza del Pino IM, Ibarra-Molero B, Sanchez-Ruiz JM. Lower kinetic limit to protein thermal stability: a proposal regarding protein stability in vivo and its relation with misfolding diseases. Proteins. 2000;40(1):58-70.
49. Robertson AD, Murphy KP. Protein Structure and the Energetics of Protein Stability. Chem Rev. 1997;97(5):1251-68.
50. Pey AL, Rodriguez-Larrea D, Bomke S, Dammers S, Godoy-Ruiz R, Garcia-Mira MM, et al. Engineering proteins with tunable thermodynamic and kinetic stabilities. Proteins. 2008;71(1):165-74.

51. Munoz V, Sanchez-Ruiz JM. Exploring protein-folding ensembles: a variable-barrier model for the analysis of equilibrium unfolding experiments. Proc Natl Acad Sci U S A. 2004;101(51):17646-51.
52. Sanchez-Romero I, Ariza A, Wilson KS, Skjot M, Vind J, De Maria L, et al. Mechanism of protein kinetic stabilization by engineered disulfide crosslinks. PLoS One. 2013;8(7):e70013.
53. Hosszu LL, Baxter NJ, Jackson GS, Power A, Clarke AR, Waltho JP, et al. Structural mobility of the human prion protein probed by backbone hydrogen exchange. Nat Struct Biol. 1999;6(8):740-3.
54. Baldwin RL, Frieden C, Rose GD. Dry molten globule intermediates and the mechanism of protein unfolding. Proteins. 2010;78(13):2725-37.
55. Georgescu RE, Li JH, Goldberg ME, Tasayco ML, Chaffotte AF. Proline isomerization-independent accumulation of an early intermediate and heterogeneity of the folding pathways of a mixed alpha/beta protein, Escherichia coli thioredoxin. Biochemistry. 1998;37(28):10286-97.
